# Linking Grassland Canopy Structure and Function Responses to Field Experimental Drought Both Seasonally and Interannually

**DOI:** 10.1002/ece3.73447

**Published:** 2026-04-16

**Authors:** Mariela Encarnación Ojeda, Xiangming Xiao, Emily U. Nguyen, Lara Souza

**Affiliations:** ^1^ School of Biological Sciences University of Oklahoma Norman Oklahoma USA; ^2^ Oklahoma Biological Survey Norman Oklahoma USA

**Keywords:** canopy function, canopy structure, drought, precipitation shifts, remote sensing

## Abstract

Shifts in species composition in water‐limited ecosystems such as temperate prairies during summer droughts can lead to community‐level changes. In both short‐term experimental and long‐term drought events, C4 species decline in abundance while C3 species increase, causing reductions in above ground net primary productivity (ANPP). Thus, this study focused on linking experimental drought‐induced shifts in species composition with changes in canopy structure and function using remote sensing approaches. We took advantage of a long‐term experimental precipitation study in Central Oklahoma that has established a gradient of seven levels of precipitation in a fully factorial randomized block design: −100%, −80%, −60%, −40%, −20% rainfall exclusion, 0% change in precipitation (i.e., control) and precipitation addition +50%. Our aim was to answer the following questions: (1) How does an experimental precipitation gradient impact canopy spectral reflectance both seasonally and interannually? (2) How does canopy structure and plant species composition vary seasonally and interannually along an experimental precipitation gradient? (3) Can we link variation in canopy reflectance to canopy structure and plant species composition under experimental drought both seasonally and interannually? Our results showed that variation in seasonal and interannual precipitation can be observed in the near‐infrared (NIR) portion of the electromagnetic spectrum. A drier year has lower normalized vegetation index (NDVI) values and higher leaf area index (LAI) values due to reduced canopy greenness and increased plant litter, respectively, due to the limited water availability. High abundance of C3 species such as *Lespedeza cuneata* that have advantageous traits can mediate canopy responses to drought. Thus, species specific abundance, in this case the high abundance of *L. cuneata* , can influence canopy reflectance. Future studies should focus on understanding the impacts of resource allocation to canopy architecture as well as the relationship between leaf traits and canopy response and how these affect canopy reflectance.

## Graphical abstract

Shifts in species composition in water‐limited ecosystems such as temperate prairies during summer droughts can lead to community‐level changes. Our study focuses on linking experimental drought‐induced shifts in species composition with changes in canopy structure and function using remote sensing approaches. We show that the effects of variation in seasonal and interannual precipitation can be observed in canopy reflectance and structure.

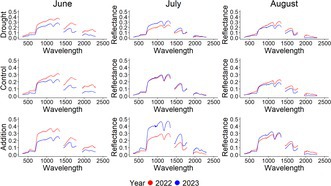

## 1 Introduction

Grasslands are dominant terrestrial biomes comprising 40% of the total land area (Petermann and Buzhdygan 2021) and are found in every region of the globe (Maestre et al. 2021). They are water‐limited ecosystems, and the seasonality of precipitation shapes the spatial–temporal dynamics in the dominance of grass and woody vegetation (Sala et al. 2017; Winkler et al. 2019). Recent studies demonstrate that drought conditions cause shifts in C3:C4 grassland species composition across both long‐term natural and short‐term experimental conditions, leading to the decline of C4 graminoids and a concurrent increase in dominance of C3 graminoids and/or forbs (Castillioni et al. 2020; Knapp et al. 2020). Precipitation seasonality shifts, particularly in drought years with lower rainfall during the summer months, have become more pronounced in the Southern Great Plains (SGP) (Bukovsky et al. 2017; Hajek and Knapp 2022). Thus, in a grassland system where water is a limiting factor, precipitation shifts can cause significant changes (Carroll et al. 2021) contributing towards the decline of C4 species and a reduction in aboveground net primary productivity (ANPP) (Knapp et al. 2020; Smith et al. 2024). Hence, by tracking and linking species composition shifts through shifts in canopy structure and function, one can understand ecosystem responses as well as feedback to drought and other climatic factors (Roberts et al. 2004; Mariotte 2014).

Remote sensing, including leaf and canopy spectrometry (spectroscopy), allows for rapid documentation of the biophysical properties of vegetation and can be used to infer functional, chemical, and morphological traits at various spatial scales (Roberts et al. 2004; Cavender‐Bares et al. 2017; Shiklomanov et al. 2019). Pigments—chlorophyll, anthocyanins, and carotenoids—absorb light in the visible range (VIS, 400–700 nm), while leaves scatter it in both the near‐infrared range (NIR, 700–1000 nm) and short‐wave infrared (SWIR, 1100–2500 nm). Water, lignin, cellulose, and phenolics create distinct species‐specific absorption patterns. Researchers have linked the normalized difference vegetation index (NDVI) (Wang et al. 2016) and the NIR (Ollinger 2011; Wang et al. 2018) to shifts in plant species composition, demonstrating that remotely sensed data can reveal canopy structure. A direct use of NDVI is to characterize canopy function which has led many studies to compare or correlate it with the leaf area index (LAI) as NDVI and NIR can be influenced by vegetation structure ‐ (i.e., closed or open canopies) (Sripada et al. 2005; Darvishzadeh et al. 2008; Xue and Su 2017; Darvishzadeh et al. 2011).

Canopies are composed of multiple plant species which, in turn, vary in their abundance and functional traits. Previous research has demonstrated that dominant species (Cheng et al. 2021) and/or dominant functional groups (Griffin‐Nolan et al. 2019) can influence community‐level trait expression more than subdominant and transient species, suggesting that their combined higher abundance and functional traits can have large impacts on ecosystem structure and function. The mass ratio hypothesis states that ecosystem functioning, at a given point in time, is determined by the trait values of the dominant contributors to plant biomass (Grime 1998; Diaz et al. 2007) and as such species composition can determine drought sensitivity (Slette et al. 2023), meaning that the traits of dominant species can determine how a plant community responds to drought. The spectral or optical diversity hypothesis suggests that species within a community occupy unique spectral spaces delineated by their chemical, anatomical, and morphological characteristics (Asner and Martin 2009; Ustin and Gamon 2010; Schweiger et al. 2018). Thus, a diverse canopy can have high spectral variation (Torresani et al. 2024). Variation in canopy reflectance is known to be affected by variation in the amount of vegetation cover (Wang et al. 2016), and as vegetation cover changes, so does spectral diversity (the number of different spectra) (Schweiger et al. 2018). The overall stability of grassland composition and ANPP under variable precipitation conditions is dependent on dominant species' relative abundance as well as functional traits (Polley et al. 2013; Shi et al. 2016). Subdominant species' responses, increases or decreases, can also impact a community's sensitivity to drought (Mariotte 2014) and in our grassland system subdominant species (C3 forbs) have increased while dominant species (C4 grasses) have declined with increasing drought (Castillioni et al. 2020).

Differences in plant performance can be due to both abiotic (soil moisture, soil temperature, etc.) and biotic (neighborhood structure) factors (Castillioni, Newman, et al. 2022). Drought has been found to impact phenology in this system by causing changes in flowering timing (earlier bloom time for some and delayed for other species) overall, leading to divergence in phenological strategies (Castillioni, Newman, et al. 2022) and impacting grassland arthropods by decreasing both abundance and diversity by increasing surface temperatures, reducing soil moisture and increasing desiccation risks (Prather et al. 2020). Drought can also have physiological impacts such as decreases in ANPP due to stomatal closure, decreased photosynthetic rates, and decreased growth (Scott et al. 2009; Keen et al. 2021). Because summer precipitation has been decreasing (i.e., summer droughts are increasing in frequency) in the SGP (Hajek and Knapp 2022), it is imperative to understand how dominance patterns, abundance, shift in short and long‐term periods and under different precipitation regimes.

This study takes advantage of a precipitation gradient established as part of the international Drought‐Net experimental network (https://droughtnet.weebly.com/) addressing grassland sensitivity to drought. Our aim is to answer the following questions: (1) How do increases and declines in precipitation impact canopy spectral reflectance (function) across the growing seasons and between years? (2) How does canopy structure (Leaf Area Index—LAI) and plant composition (plant diversity, evenness, richness, and plant species composition) vary over time (across seasons and between years) along an experimental precipitation gradient? (3) Can we link variation in canopy function to canopy structure and plant species composition? We predicted (1) that canopy level reflectance would decrease in the visible (VIS) due to a reduction in photosynthetic pigments and increase in the near‐infrared (NIR) portion of the reflectance curve under experimental drought, as the growing season progresses, and in drier years. We also predicted (2) shifts in plant species composition through changes in species' ‐dominance (evenness) rather than changes in species numbers (richness), to reduce canopy structure (LAI) and function (spectral response) under experimental drought, as the growing season progresses, and in drier years. Finally, we predicted that (3) canopy structure (LAI) and function (NDVI and NDWI) would be positively associated under higher water availability (increasing “nonsenesced” leaf area promotes canopy function) and negatively associated (increasing “senesced” leaf area reduces canopy function) under periods of drought.

## 2 Materials and Methods

### 2.1 Study Site

This study was conducted at Kessler Atmospheric and Ecological Field Station (KAEFS; 34.981, −97.532) in Central Oklahoma, in a mesic temperate mixed‐grass prairie dominated by C4 and C3 graminoids, forbs and nitrogen fixers (N‐fixers) (Buthod and Hoagland 2016; Castillioni et al. 2020; Castillioni, Newman, et al. 2022; Castillioni, Patten, and Souza 2022). The most dominant species that encompass about 70% of the total vegetation cover are: *Schizachyrium scoparium, Sorghastrum nutans, Sporobolus compositus* (C4 grasses), *Dicanthelium oligosanthes* (C3 grass), *Symphyotrichum ericoides, Croton monanthogynus, Calylophus serralatus, Erigeron strigosus* (C3 forbs), *Desmanthus illinoensis, Dalea purpurea* (N‐fixers) as well as the nonnative or invasive species *Bothrichloa ischaemum* (C4) and *Lespedeza cuneata* (N‐fixers). KAEFS was abandoned from field cropping in 1973 and contains encroaching *Juniperus virginiana, Rhus copallinum*, and *Rhus glabra* (Castillioni et al. 2020; Castillioni, Newman, et al. 2022). The overall site is located within the subtropical humid climate zone and has a mean annual temperature of 16°C, with the lowest temperatures occurring in January and the highest in July (Trewartha 1968; Oklahoma Climatological Survey 2016). The average yearly precipitation for the site is 914.6 mm. The site's soil belongs to the Nash‐Lucien complex and exhibits a neutral pH, high water‐holding capacity, and a moderately penetrable root zone (Xu et al. 2013; Castillioni et al. 2020).

### 2.2 Experimental Design

The site is part of a long‐term drought experiment established in 2016 (Castillioni et al. 2020; Castillioni, Newman, et al. 2022; Castillioni, Patten, and Souza 2022) as part of the Drought Net, a global experimental network (Drought‐Net: http://wp.natsci.colostate.edu/droughtnet/; Smith et al. 2024). We have experimentally manipulated precipitation in the field by randomly assigning seven levels of precipitation in a fully factorial randomized block design: −100%, −80%, −60%, −40%, −20% rainfall exclusion, 0% change in precipitation (i.e., control) and precipitation addition +50%. Each treatment had 3 replicates for a total of 21 plots. We subdivided each experimental plot (4 m × 4 m) into four 1 m × 1 m subplots: one clipped to mimic hay harvest, one designated for plant composition and phenology, one for monitoring aboveground production, and one for collecting net CO_2_ exchange, soil respiration, and belowground production (Castillioni et al. 2020; Castillioni, Newman, et al. 2022; Castillioni, Patten, and Souza 2022). All plots have rain interception shelters made of acrylic transparent plastic that block rain and allow > 93% of solar radiation, including the control plots to exclude confounding effects of shelter presence (Castillioni et al. 2020; Castillioni, Patten, and Souza 2022). In the 0% control treatment the panels are downward facing allowing all precipitation to fall into the plot, within the reduction treatments rainwater is diverted from the plots depending on how the shelter panels were arranged (upwards and/or downwards) to meet the precipitation reduction percentage of each treatment and in the addition plots additional panels on either side two sides of the plot divert additional precipitation onto the plot (Castillioni et al. 2020; Castillioni, Newman, et al. 2022; Castillioni, Patten, and Souza 2022). The width of each additional panel sheet was 25% the width of the experimental plot, together equaling 50% of the plot (Castillioni et al. 2020; Castillioni, Newman, et al. 2022; Castillioni, Patten, and Souza 2022). We collected all observations from the 1 m × 1 m subplot designated for plant composition and phenology.

### 2.3 Canopy Reflectance

Remote sensing is based on the measurement of reflected radiation from different bodies, in this case the amount of solar radiation that vegetation reflects into the atmosphere. We acquired canopy‐level spectral reflectance measurements using an ASD FieldSpec3, which is a hyperspectral sensor that collects data in the full spectrum (350 nm–2500 nm), for the summer growing season, from June to August, of 2022 and 2023. We selected a hyperspectral sensor because it produces data that can be statistically linked to vegetation characteristics and consists of high‐dimensional, fine spectral bands that are highly correlated with each other (Landgrebe 2002; Ling et al. 2019). Our measurements were done at least once per month at 100 cm from the ground. The measurements were conducted between 9 am and 4 pm with white reference calibrations when necessary and during clear sunny days or partly cloudy days if no clear days occurred within the time of collection. To preserve the integrity of our experimental design, we only measured in three treatments, 0% (control), −100% (severe drought), and +50% (precipitation addition) because data collection required panel removal to avoid noise caused by scattering (*n* = 3 replicates, *N* = 9 total plots).

To visualize reflectance curves, we removed areas of noise (e.g., water absorption bands and scattering) and applied Savitzky–Golay (SG) filter to smooth the curves while preserving the shape using the package “prospectr” (Stevens and Ramirez‐Lopez 2025). The Normalized Difference Vegetation Index (NDVI: [800–680/800 + 680]) and the Normalized Difference Water Index (NDWI: [857–1241/857 + 1241]) were calculated from the acquired reflectance. These indices were chosen because they are common and effective methods of estimating vegetation health (i.e., greenness), biomass, and water content. Additionally, we used the Holland Scientific RapidSCAN CS‐45 to acquire NDVI (further referred to as CropScan NDVI) for all plots (*N* = 21) as this measurement does not require rain shelter removal because it has its own light source. The RapidSCAN CS‐45 has an internal polychromatic light source and three spectral bands (670 nm, 730 nm, and 780 nm) for acquiring NDVI (780–670/780 + 670) measurements (Holland Scientific). For each plot, two measurements were collected and then averaged into a single measurement. We conducted these measurements biweekly.

### 2.4 Canopy Structure

We used the Accu‐PAR‐LP‐80 Ceptometer to acquire the Leaf Area Index (LAI), a biophysical parameter related to structure that can determine processes like photosynthesis, canopy water interception and biomass production (Chen and Cihlar 1996; Broge and Mortensen 2002; Abdel‐Rahman et al. 2014; Asam et al. 2015; Kiala et al. 2017). The Accu‐PAR‐LP‐80 Ceptometer uses the photosynthetic active radiation (PAR) inversion technique to calculate LAI (METER Group). For each plot (*N* = 21), we collected two measurements and averaged them into a single value. LAI was measured once every 2 weeks.

### 2.5 Plant Composition

To determine the effects of drought and year on total plant cover, plant diversity, and species composition, we tracked the identity of plant species and species‐specific foliar cover at the subplot level twice in the growing season (May and July) across both 2022 and 2023. We used a 1 m x 1 m quadrat and only assessed individuals that were rooted within the quadrat. We assigned foliar cover using a modified Braun‐Blanquet scale with six categories: 1 = < 1%, 2 = 1%–5%, 3 = 5%–25%, 4 = 25%–50%, 5 = 50%–75%, and 6 = 75%–100%. We quantified species richness, evenness, and diversity (Shannon's Diversity Index) from species‐specific foliar monthly cover data by using the median of each cover class category as our abundance value and then using the DIVERSE function in PRIMER‐e v. 6 software (www.primer‐e.com; Anderson et al. 2008).

### 2.6 Abiotic Factors: Precipitation & Soil Moisture

The Standardized Precipitation Index (SPI) is a drought index that is widely used for drought detection as it measures normalized anomalies in precipitation (McKee et al. 1993; Guttman 1999; Stagge et al. 2015). SPI is able to detect drought at different time scales (1, 3, 6, 12 and 24 months), implying that distinct types of droughts (meteorological, agricultural, and hydrological) can be monitored (Tirivarombo et al. 2018). We calculated SPI at 1‐month, 3‐month and 6‐month using precipitation data from 1994 to 2023 and the R package “SPEI” R (v. 4.3.2) (Beguería and Vicente‐Serrano 2023). We focused on the values for the spring and summer seasons of 2022 and 2023. We acquired site‐level monthly precipitation data from the Washington (WASH) Mesonet Station located in KAEFS. Since SPI is sensitive to the quantity and reliability of the data used to fit the distribution, we used 29 years of data (Keyantash et al. 2023; McKee et al. 1993). SPI values are considered extremely dry if < −2.0, severely dry if −2.0 < SPI < −1.5, moderately dry −1.5 < SPI < −1.0, near normal if ≤ −0.5 and/or ≤ 0.5, moderately wet if 1.0 < SPI < 1.5, very wet if 1.5 < SPI < 2.0 and extremely wet if < 2.0.

### 2.7 Data Analyses

#### 2.7.1 Canopy Reflectance, Structure & Plant Composition Metrics

To determine the impacts of experimental drought overtime on canopy function (Question 1), we acquired the area under the curve (AUC) for all spectral vegetation curves for the growing seasons (May, June, July) of 2022 and 2023. To calculate the AUC, we divided the curves into three sections: 400–700 nm (visible), 700–1100 nm (NIR), and 1100–2500 nm (SWIR) (Cavender‐Bares et al. 2017). We only partially used the SWIR section because noise absorption and sensor errors produced reflectance values of zero. Additionally, we focused on three spectral regions that capture the water absorption reflectance troughs (970 nm, 1175 nm, and 1450 nm). We used KaleidaGraph to calculate AUCs (KaleidaGraph, Version 4.5.4 for Windows. Synergy Software, Reading, PA, USA. www.synergy.com). To assess how the effects of experimental precipitation, seasonal and interannual variation in background precipitation as well as their potential interactions influenced canopy spectral reflectance (Question 1), we conducted analyses of variance (ANOVA) in R (v. 4.3.2) and post hoc Tukey HSD test using the “rstatix” package (Kassambara 2023). Because we only measured in 3 treatments, 0% (control), −100% (severe drought), and + 50% (precipitation addition), this analysis consisted of a total of 9 plots across all three blocks for a total of 54 observations for each test.

However, to better understand how drought impacted canopy reflectance—CropScan NDVI—overtime (Question 1), we used a linear mixed‐effects model. To address how experimental drought impacted canopy structure (LAI) (Question 2), we used linear mixed‐effects models containing the “lme4” package (Bates et al. 2015) in R across all plots (*N* = 21), not just the extremes (0%, −100 & +50). Our linear mixed‐effects model used the following variables: precipitation treatment (fixed), plot (fixed), block (random), month (random), and year (random) to address how precipitation affected canopy structure across the summer growing seasons over 2 years. Both block and plot were treated as random factors in the model to account for uncontrolled variation (Castillioni et al. 2020). From the linear mixed‐effects model, an ANOVA was conducted using the “car” package.

To determine how experimental drought affected plant community metrics (Question 2), we fit generalized linear models (GLM) to assess the impact of precipitation, seasonal and interannual variation as they require links to establish distribution instead of assuming normality. For species richness the GLM was conducted with a Poisson distribution and a log link as is done for count data, and for evenness it was done with a Gamma distribution with an inverse link in R using the “stats” package (Castillioni et al. 2020; Warton et al. 2015). We conducted a goodness of fit test using the “aods3” package (Lesnoff and Lancelot 2024), which evaluates the accuracy of the chosen model representing the observed data. We then used the GLM models in an ANOVA using the “car” package (Fox and Weisberg 2019) using the following plant metrics: richness, evenness and diversity. For diversity, we fit a linear model and conducted the ANOVA in the same way as previous using treatment (fixed effect), month, year, or their interaction (random effects) using the “car” package. We performed a post hoc pairwise comparison test using the “emmeans” package to identify differences among precipitation treatments for evenness and diversity. We used this approach as Tukey HSD assumes raw‐scale means are normally distributed, which can misrepresent effects for GLMs.

#### 2.7.2 Nonparametric Permutation Analyses & Conditional Inference Trees

To test for experimental precipitation treatment and variation in precipitation overtime effects on plant composition (Question 2), we used a nonparametric permutational multivariate analysis of variance (PERMANOVA) in PRIMER‐e v.6 (Anderson 2001). The PERMANOVA constructed an ANOVA‐like test statistic from a dissimilarity matrix using Bray‐Curtis's similarity and calculated a *p*‐value using random permutations of observations among groups (Anderson and Walsh 2013). We conducted PERMANOVA pairwise tests to understand which blocks differed across years and applied a Bonferroni correction (Table S1). All PERMANOVA were based on 9999 restricted permutations. We performed a similarity percentage analysis (SIMPER) to identify the most abundant species within each treatment, using untransformed data (Gioria and Osborne 2009).

To identify factors that influenced canopy structure (LAI) and function (CropScan NDVI), we used the function “ctree” from the package “partykit” (Borkovec and Madin 2019) in R to generate conditional inference trees (Question 2). Conditional Inference Trees estimate a regression relationship by binary recursive partitioning in a conditional inference framework and, as such, are a useful approach for nonparametric and unbalanced datasets. We generated the Conditional Inference Trees that contained both abiotic and biotic predictors. Our abiotic variables were: plot number, block, month of data collection, year, precipitation treatment categories (all seven treatments), broader precipitation categories (wet, control, and dry), site‐level rainfall, and all SPI levels (1‐, 3‐, and 6‐month). Our biotic variables were: plant evenness, richness, diversity, the overall presence of *Lespedeza cuneata*, as well as the early and late season presence of *L. cuneata*, (dominant C3 legume), and the presence of *Bothrichloa ischaemum* (a dominant C4 graminoid). We focused on these two species because their abundance may influence canopy responses, as strong environmental perturbations can shift competitive advantages to new species (Tilman et al. 1997; Wilfahrt et al. 2023).

#### 2.7.3 Correlational Analyses Between Canopy Structure and Function

To address the linkages between canopy structure and canopy function across experimental precipitation overtime (Question 3) we used Spearman correlations in R. Specifically, Spearman correlations allowed us to assess if there was a significant relationship between the CropScan NDVI and LAI (*N* = 21) across treatments, seasonally and temporally. As well as assess relationships between LAI, NDVI and NDWI, calculated from the reflectance curves for the precipitation gradient extremes (*N* = 9).

#### 2.7.4 Overall Analytical Framework

Across our three research questions, we used complementary statistical approaches matched to the structure and distribution of each dataset and to the level at which observations were collected. For Question 1 (canopy function), ANOVAs were applied to the spectral AUC values because this subset included only the extreme precipitation treatments (0%, −100%, +50%) and produced a balanced dataset. Each spectral curve represented one measurement per plot × month × year. We decided to use an ANOVA since the AUC allowed us to simplify specific regions of the spectral curve with a single scalar value. For NDVI measured with the RapidSCAN (all seven treatments; all 21 plots) we used linear mixed‐effects models instead of an ANOVA due to this being a more robust and complex dataset as well as the fact that LME models handle repeated measures. Thus, the observational units were plot × block × treatment × month × year. Each observation represented the average NDVI for a plot per month × year. For Question 2 (canopy structure and plant composition), LAI was analyzed with mixed‐effects models similarly to NDVI. Plant richness, evenness, and diversity were modeled with GLMs because the data violated normality (count or strictly positive distributions). The overall observational units were treatment × month × year and each observation constituted a quadrat matrix. Multivariate plant composition was analyzed using PERMANOVA and SIMPER with their observational units being treatment × month × year × plot × block. For Question 3 (linking structure and function), we used Spearman correlations to evaluate associations between LAI and spectral indices and we used conditional inference trees to identify both biotic and abiotic drivers of LAI and NDVI because this approach accommodates nonnormal predictors and unbalanced datasets. Together, this integrated framework allowed us to (1) isolate the effects of precipitation, season, and year on individual response variables, (2) evaluate multivariate changes in species composition, and (3) link structural and functional responses while accounting for repeated measures, nested design, and species‐specific effects.

## 3 Results

### 3.1 Interannual Canopy Reflectance

**FIGURE 1:**
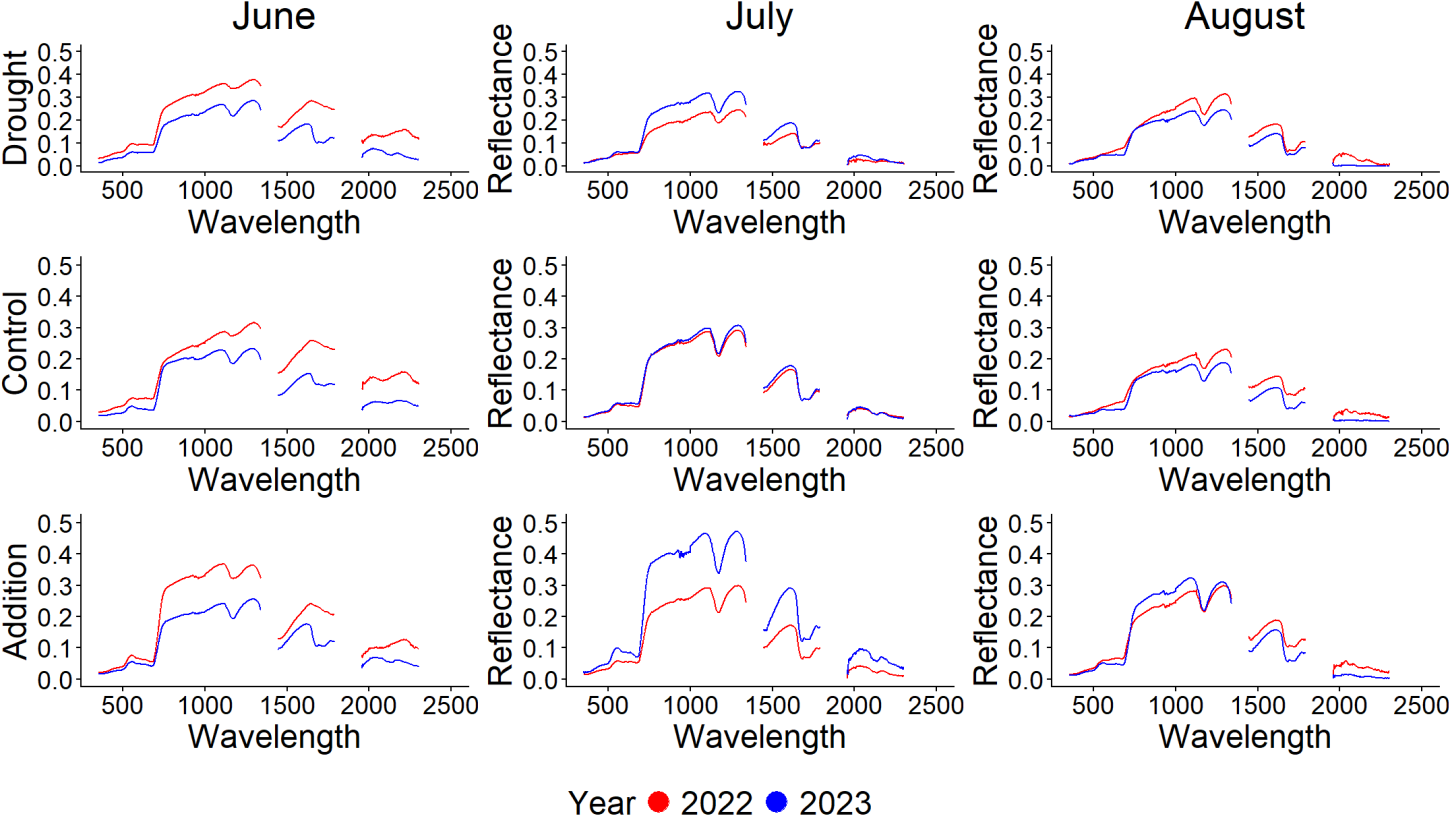
Reflectance curves for the precipitation extremes across the summer growing season (Left Panel: June, Middle Panel: July, Right Panel: August) for both 2022 and 2023. Drought, control, and precipitation addition treatments are represented in top, middle, and bottom panels, respectively. All areas containing noise were removed and a Savitzky–Golay (SG) filter was applied.

Grassland canopy reflectance was reduced under drought treatments, specifically at the end of the summer growing season, and in the drier year (2022) (Figures 1 and S3). Canopy reflectance varied interannually and across our precipitation gradient extremes (0%, +50% and −100). Canopy spectral responses diverged interannually at the start and end of the growing season but converged in midseason (July) (Figure 1). Canopy NIR region reflectance differed significantly in precipitation addition treatments from both control and drought plots (*p* = 0.013, Table 1). Canopy NIR was 27% greater in precipitation addition plots than drought and control plots indicating that those plots may have experienced stress, which can cause reduced water content or damage to cell structures. However, when observing both spectra together (400–1100 nm) NIR was no longer influenced by year (*p* = 0.082, Table 1), and treatment was the only significant difference (*p* = 0.013, Table 1) observed. In the SWIR portion of the spectrum (1463‐1774 nm), month (*p* = 0.0008, Table 1) and the interaction of month and year (*p* = 0.023, Table 1) were significant, with June having the overall highest AUC and August the lowest (26% and 41% respectively) indicating low canopy water content early and high canopy water content later in the season. However, we did not detect precipitation treatment. Furthermore, canopy SWIR AUC was 20% higher in June 2022 than in 2023, 30% higher in July 2023 than in 2022 and 29% higher in August 2022 than in 2023, indicating that seasonality has differing impacts on canopy water/function in dry (2022) versus mesic (2023) years. When focusing on the water absorption troughs (Jiang and Carrow 2007; Caturegli et al., 2020) in the SWIR, we found that at 1175 nm there is a significant month difference (*p* = 0.013, Table 1) in reflectance where June had 29% higher reflectance in that wavelength than August showing the same pattern of low canopy water content early and high canopy water content later in the season.

**TABLE 1:** Analyses of Variance (ANOVA) conducted for all responses (Visible, Near‐Infrared, SWIR) testing for the effects of treatment, month, year, and their interactions.

ANOVA table			
Response	Effect	*F*‐statistic	*p*
AUC Visible Wavelength	Treatment	2.427	0.104
	Month	2.573	0.092
	Year	3.928	0.056
	Treatment:Month	2.356	0.074
	Treatment:Year	0.31	0.736
	Month:Year	0.9	0.416
	Treatment:Month:Year	0.021	0.999
AUC Near‐Infrared Wavelength	Treatment	4.958	0.013*
	Month	1.915	0.163
	Year	0.064	0.802
	Treatment:Month	2.009	0.116
	Treatment:Year	0.05	0.951
	Month:Year	0.419	0.661
	Treatment:Month:Year	0.164	0.955
AUC Visible & Near‐Infrared Wavelength	Treatment	5.01	0.013*
	Month	2.095	0.139
	Year	0.258	0.615
	Treatment:Month	2.275	0.082
	Treatment:Year	0.051	0.95
	Month:Year	0.515	0.602
	Treatment:Month:Year	0.133	0.969
AUC Shortwave Infrared Wavelength	Treatment	2.23	0.123
	Month	8.834	0.0008*
	Year	0.485	0.491
	Treatment:Month	1.017	0.413
	Treatment:Year	0.364	0.697
	Month:Year	4.25	0.023*
	Treatment:Month:Year	0.301	0.875
1175 nm Reflectance Trough	Treatment	0.275	0.761
	Month	4.995	0.013*
	Year	0.631	0.433
	Treatment:Month	1.255	0.307
	Treatment:Year	2.074	0.141
	Month:Year	1.51	0.235
	Treatment:Month:Year	0.847	0.505
1450 nm Reflectance Trough	Treatment	0.334	0.718
	Month	2.918	0.068
	Year	4.008	0.053
	Treatment:Month	0.499	0.737
	Treatment:Year	0.958	0.394
	Month:Year	1.348	0.273
	Treatment:Month:Year	1.171	0.341
970 nm Reflectance Trough	Treatment	1.522	0.233
	Month	1.7	0.198
	Year	0.134	0.717
	Treatment:Month	1.463	0.235
	Treatment:Year	2.331	0.113
	Month:Year	0.427	0.656
	Treatment:Month:Year	0.934	0.456

*Note:* Statistically significant values have asterisks added.

Abbreviation: AUC, area under the curve.

### 3.2 Drought Impacts on Ecosystem Function Over Time

**FIGURE 2:**
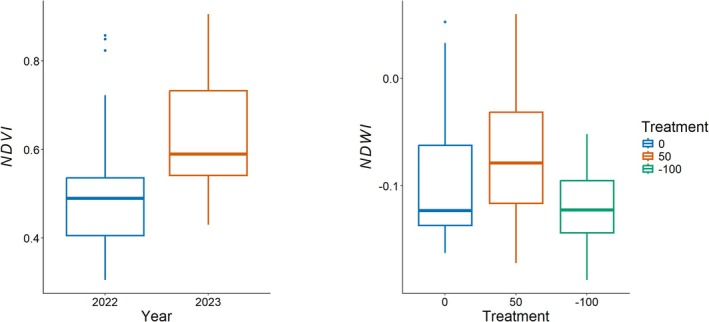
(A) Interannual (2022 vs. 2023) Normalized Difference Vegetation Index (NDVI) for the extreme plots. (B) Normalized Difference Water Index (NDWI) across extreme plots (*N* = 9; Treatments: Control (0%), Addition (+50%) and Drought (−100%)).

Vegetation indices were higher in wetter years than in drier years. Both NDWI and NDVI, for the extreme treatments (*N* = 9; 0%, +50% and −100%) showed higher values for 2023 indicating that plants were greener and had greater water content (Figure 2A,B). However, only NDVI showed significant interannual differences (*p* = 0.007, Table 2) with mean NDVI in 2023 being 17% higher than 2022. NDWI showed treatment differences (*p* = 0.040, Table 2) instead, with the severe drought treatments being 41% lower than the control and addition plots. The timing of precipitation impacts greenness; wetter years tend to be greener than drier years and the spring growing season may impact the summer growing season.

**TABLE 2:** Analyses of Variance (ANOVA) for canopy function and structure factors testing for the effects of treatment, month, year, and their interactions.

ANOVA table			
Response	Effect	*F*‐statistic	*p*
NDVI (Extremes; *N* = 9)	Treatment	2.672	0.084
	Month	0.907	0.413
	Year	8.205	0.007*
	Treatment:Month	0.573	0.684
	Treatment:Year	0.491	0.617
	Month:Year	1.031	0.368
	Treatment:Month:Year	0.261	0.901
NDWI (Extremes; *N* = 9)	Treatment	3.546	0.040*
	Month	0.064	0.938
	Year	1.657	0.207
	Treatment:Month	0.451	0.771
	Treatment:Year	1.227	0.306
	Month:Year	2.359	0.110
	Treatment:Month:Year	0.632	0.643
CropScan NDVI (All plots; *N* = 21)	Treatment	2.879	0.012*
	Month	25.203	1.96e‐06*
	Year	14.77	3.61e‐08*
	Treatment:Month	0.642	0.696
	Treatment:Year	0.456	0.971
	Month:Year	4.356	0.006*
	Treatment:Month:Year	0.565	0.917
LAI (All plots; *N* = 21)	Treatment	0.395	0.677
	Month	18.498	1.24e‐04*
	Year	10.065	3.37e‐04*
	Treatment:Month	0.072	0.93
	Treatment:Year	3.16	0.025*
	Month:Year	0.684	0.511
	Treatment:Month:Year	2.04	0.109
CropScan NDVI (Extremes; *N* = 9)	Treatment	2.866	0.067
	Month	7.79	0.008*
	Year	3.329	0.027*
	Treatment:Month	0.272	0.763
	Treatment:Year	0.154	0.987
	Month:Year	1.55	0.214
	Treatment:Month:Year	0.076	0.998
LAI (Extremes; *N* = 9)	Treatment	0.205	0.815
	Month	9.631	0.004*
	Year	0.55	0.581
	Treatment:Month	0.038	0.963
	Treatment:Year	0.102	0.981
	Month:Year	1.145	0.329
	Treatment:Month:Year	0.242	0.913

*Note:* Statistically significant values have asterisks added.

**FIGURE 3:**
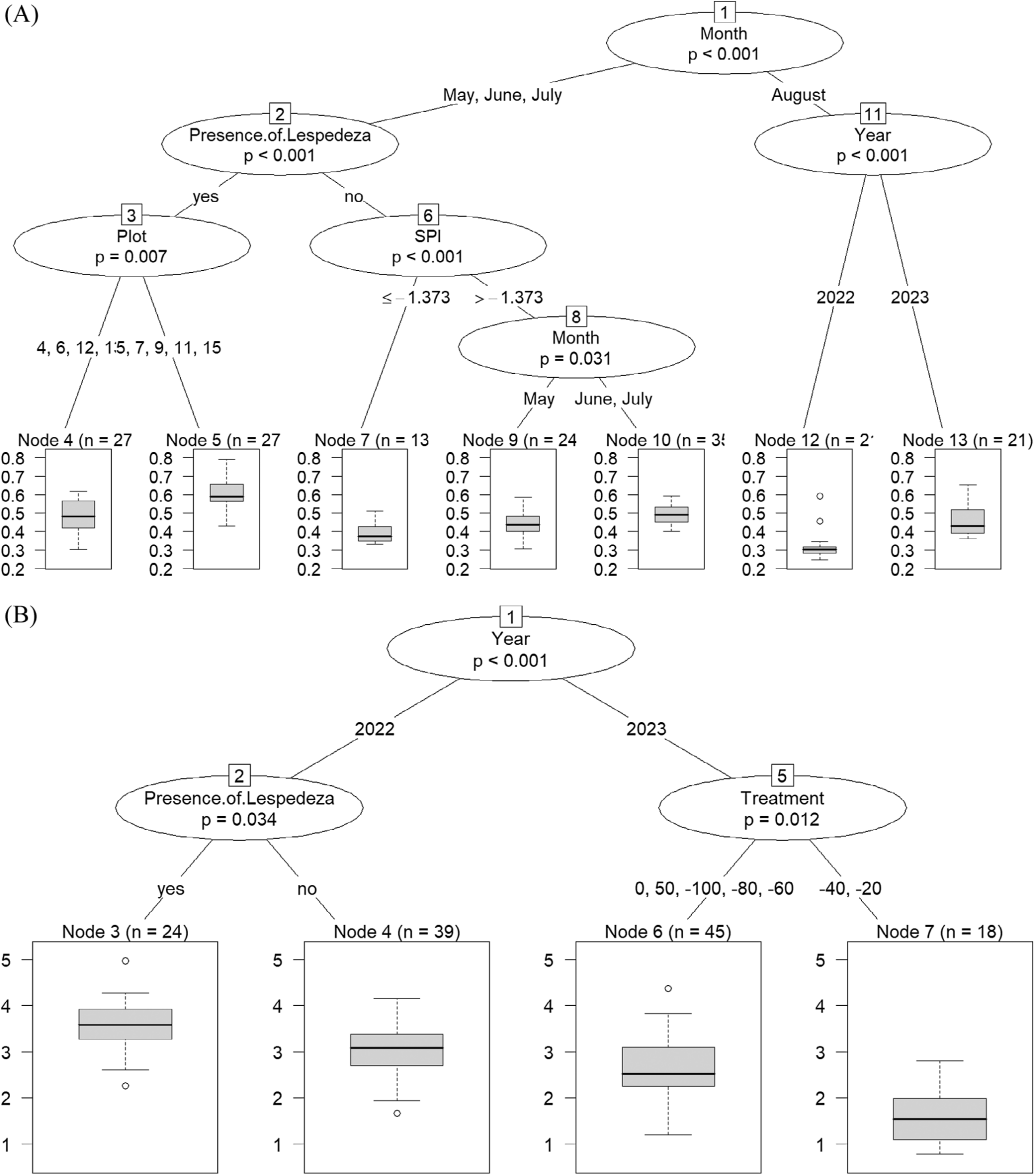
(A) Conditional Inference Tree for CropScan NDVI with both biotic and abiotic factors for all plots (*N* = 21). (B) Conditional Inference Tree for LAI with both biotic and abiotic factors for all plots (*N* = 21). Each node indicates data separation and boxplots note values.

CropScan NDVI, for all treatments (*N* = 21) varied by month (*p* = 6.43e‐07, Table 2), by the interaction of treatment and year (*p* = 5.79e‐04, Table 2), and by the interaction of month and year especially early in the season (May) (*p* = 2.12e‐02, Table 2). The Conditional Inference Tree generated with both biotic and abiotic factors showed that the overall presence of *Lespedeza cuneata* , as well as its presence later in the season, promoted higher NDVI values in our mesic year (2023) but not in our dry year (2022) (Figure 3A).

### 3.3 Drought Impact on Canopy Structure Over Time

The presence and dominance of a C3 species, *Lespedeza cuneata* , mediated seasonal and interannual responses of canopy structure (LAI) and function (NDVI) to variation in precipitation. Only during a dry year, the presence of *Lespedeza* promoted canopy structure while in a mesic year this effect did not take place (Figure 3B). In fact, during our dry year LAI increased in our experimental drought treatments (*p* = 0.030, Table 2) likely due to increased senesced vegetation, as mean LAI for 2022 was 28% higher than in 2023. However, our mesic year exhibited treatment differences (*p* = 0.012, Table 2).

### 3.4 Drought Effects on Plant Composition Over Time

Drought promoted plant evenness—reduced dominance of C4 species—while drier periods reduced diversity (Table 3). Plant evenness was impacted by treatment (*p* = 1.03e‐13), year (*p* = 0.0461), the interaction between treatment and block (*p* = 5.94e‐12) as well as the interaction of block and year (*p* = 0.0357). The control and water addition treatments differed from each other, with the addition plots having lower evenness. Additionally, the water addition plots had significantly lower evenness than all of the drought plots except for the 80% reduction plots (see Table S1). The 80% reduction plots differed from the severe drought plots (−100%) with 80% reduction treatments having lower evenness. Diversity was found to have significant differences in block (*p* = 0.0035), treatment (*p* = 4.874e‐06), the interaction between year and block (*p* = 0.0386) and the interaction between block and treatment (*p* = 0.0035). The control and addition plots differed from each other, with the addition plots having lower diversity. Water addition plots had significantly lower diversity than all of the drought plots except for the 80% reduction plots (see Table S3). While the 80% reduction differed from the severe drought, 40% reduction and 20% reduction, with 80% reduction having lower mean diversity. The 40% reduction plots also had significantly higher diversity than the 20% reduction plots (see Table S3). The treatment with the highest evenness was the severe drought treatment and the treatment with the highest diversity was the 40% reduction treatment. Species richness was found not to have any significant differences or interactions across treatments.

**TABLE 3:** Analyses of variance (ANOVA) conducted for plant diversity metrics (plant richness, evenness, diversity) testing for the effects of treatment, block, year, and their interactions.

ANOVA table			
Response	Effect	Likelihood Ratio Chi‐Square	*p*
Plant richness	Year	0.3337	0.5635
	Block	1.1121	0.5735
	Year × Block	0.2643	0.8762
	Treatment	6.555	0.364
	Year × Treatment	0.8427	0.9909
	Block × Treatment	10.0207	0.6141
	Year × Block × Treatment	2.1871	0.9991

Response	Effect	*F*‐statistic	*p*
Plant diversity	Year	3.6079	0.0644
	Block	6.4796	0.0035*
	Year × Block	3.5198	0.0386*
	Treatment	8.4561	4.874e‐06 *
	Year × Treatment	0.5959	0.7318
	Block × Treatment	3.0664	0.0035*
	Year × Block × Treatment	1.0771	0.4028

Response	Effect	Likelihood Ratio Chi‐Square	*p*
Plant evenness	Year	3.979	0.0461*
	Block	4.933	0.0850
	Year × Block	6.665	0.0357*
	Treatment	72.914	1.03e‐13*
	Year × Treatment	7.594	0.2694
	Block × Treatment	79.169	5.94e‐12*
	Year × Block × Treatment	11.841	0.4586

*Note:* Statistically significant values have asterisks added.

Our PERMANOVA revealed that plant composition varied significantly across treatments, blocks, and the interactions between year and block as well as block and treatment. Our PERMANOVA pairwise test for the interaction of year and block found that composition varied interannually across plots in block 1 and 3 but not in those of block 2. Our SIMPER test showed that in water addition, control, severe drought, and 60% reduction plots. *L. cuneata* ‐ a C3 legume‐ was one of the most abundant species (Table 4). However, the test showed that our treatment replicates had low similarity to one another in terms of species composition with the lowest similarity being the addition plots (28%), and the highest similarity across replicates being the 80% reduction (65%) (see Tables 5 and S4).

**TABLE 4:** Nonparametric permutational multivariate analysis of variance (PERMANOVA) conducted for early season plant composition (May) and across the growing season (May–July) testing for the effects of treatment, block, year, and their interactions.

PERMANOVA table			
Response	Effect	Pseudo‐F	*p*
Plant composition (May & June/July)	Year	0.83258	0.5083
	Treatment	1.9975	0.0029*
	Block	17.66	0.0001*
	Year × Treatment	1.042	0.4254
	Year × Block	3.9868	0.0001*
	Treatment × Block	6.7455	0.0001*
	Year × Treatment × Block	1.0905	0.3031
Plant composition (May)	Year	0.95812	0.3574
	Treatment	2.0122	0.0024*
	Block	9.1817	0.0001*
	Year × Treatment	0.91192	0.6096
	Year × Block	3.0368	0.0093
	Treatment × Block	3.7067	0.0001*

*Note:* Statistically significant values have asterisks added.

**TABLE 5:** Similarity percentages (SIMPER) results for the top five contributing species for all treatments as well as the average similarity among replicates for all treatments.

Species	50%	0%	−20%	−40%	−60%	−80%	−100%
*Bothrichloa ischaemum*	23.48	—	20.12	—	—	—	—
*Sporobolus compositus*	21.56	11.68	—	—	18.9	75.09	10.17
*Lespedeza cuneata*	16.64	22.56	—	—	12.05	—	—
*Schizachyrium scoparium*	14.47	15.98	24.97	37.78	—	6.43	18.77
*Symphyotrichum ericoides*	13.75	13.98	27.95	17.2	32.5	5.82	9.67
*Sorghastrum nutans*	—	27.47	6.22	—	15.3	—	27.47
*Croton monanthogynus*	—	—	—	20.95	—	7.25	—
*Calylophus serralatus*	—	—	4.1	3.34	—	—	15.76
*Liatris squarrosa*	—	—	—	3.24	—	—	—
*Dichanthelium oligosanthes*	—	—	—	—	7.13	—	—

Average similarity (%)	Contribution (%)						
	50%	0%	−20%	−40%	−60%	−80%	−100%
	28.44	41.78	37.39	32.21	37.91	65.26	38.6

### 3.5 Linking Canopy Function and Structure

We found a positive relationship (*R* = 0.44, *p* = 0.048; Figure S4) between CropScan NDVI and LAI during June 2023, with higher LAI values correlated with higher NDVI values. Additionally, we found a negative relationship (*R* = −0.89, *p* = 0.033; Figure S4) between CropScan NDVI and LAI during July for our precipitation addition treatments (+50%), with lower LAI values correlated with higher NDVI values. The month of August exhibited a negative relationship (*R* = −0.056, *p* = 1.4e‐10; Figure S5). However, no significant correlation was found between the CropScan NDVI and LAI interannually nor across our precipitation gradient or interannually across all seven treatments (*N* = 21; all plots; Figure S6).

Furthermore, we found a negative relationship (*R* = −0.52, *p* = 0.027; Figure S6) between LAI and NDVI for the precipitation addition treatments. We also found a negative relationship (*R* = −0.73, *p* = 0.031; Figure S7) in 2022 between LAI and NDVI for the severe drought treatments (−100%). We found a negative seasonal relationship between LAI and NDVI during August (*R* = −0.54, *p* = 0.013). As previously mentioned, the relationship between LAI and NDVI during July for our precipitation addition treatments was negative (*R* = −0.94, *p* = 0.017). Similar to our previous results, we found no significant correlation between NDVI (calculated from reflectance; *N* = 9) and LAI interannually.

We found a negative relationship between LAI and NDWI during July for our precipitation addition treatments (*R* = −0.94, *p* = 0.017; Figure S8). Additionally, we found that during the month of August LAI and NDWI have a negative relationship (*R* = −0.54, *p* = 0.022). However, no significant correlation was found between LAI and NDWI interannually nor across our precipitation gradient extremes (*N* = 9).

## 4 Discussion

This study contributes to the understanding of grassland vegetation responses to shifting precipitation (Hoover and Rogers 2016; Hoover et al. 2018; Knapp et al. 2020; Castillioni et al. 2020; Castillioni, Newman, et al. 2022; Castillioni, Patten, and Souza 2022; Hajek and Knapp 2022), specifically drought, in the SGP which is forecasted to increase in the frequency of summer drought events (Bukovsky et al. 2017). Drought altered grassland canopy structure and function primarily through shifts in species dominance (plant evenness) rather than through changes in plant richness or diversity. Across both experimental precipitation treatments and temporal variation (seasonal & interannual) in background climate, canopy spectral responses and structural dynamics reflected changes in the presence and likely phenology of dominant species—particularly the drought‐tolerant C3 legume *Lespedeza cuneata* —rather than losses in species number. These compositional shifts mediated canopy greenness, water content, and structural layering, resulting in context‐dependent relationships between vegetation indices (NDVI, NDWI) and leaf area index (LAI).

By integrating canopy‐level spectral measurements, structural metrics, and plant community composition under experimental drought conditions across seasons and years, our results demonstrate that who dominates the canopy matters more than how many species are present for determining grassland canopy responses to drought. Plant dominance‐driven mechanism helps explain why canopy greenness and LAI can become decoupled under water limitation, particularly during late‐season droughts when senescence and litter accumulation increase. Previous studies have found that standing senesced materials can impact field LAI data collection because nonphotosynthetic vegetation also absorbs sunlight in some wavelength ranges and the AccuPAR is sensitive to all light‐blocking objects making it difficult to distinguish between senesced and photosynthetic vegetation (Klingler et al. 2020; Xu et al. 2020). Additionally, NDVI has been found to have a strong positive correlation with precipitation (Wang et al. 2003). Together, these findings highlight species‐specific trait responses and phenology as key drivers of grassland canopy function under increasingly variable precipitation regimes.

### 4.1 Precipitation and Seasonality Effects on Canopy Reflectance

We predicted that canopy level reflectance would decrease in the visible portion and increase in the NIR portion of the reflectance curve under drought conditions. However, we only found treatment differences for the AUC in the NIR region (*p* = 0.013, Table 1) Our post hoc found that addition treatments differ from both control and severe drought plots by having higher reflectance in the NIR (see Table S2). That, along with interannual variations in NDVI, suggests that drought reduces canopy greenness and that resource allocation strategies may lead to treatment differences as plants respond to drought with decreases in biomass and species‐specific responses (Schneider et al. 2014; Didiano et al. 2016). Additionally, the treatment differences may be due to differences in species composition, diversity, and evenness. The monthly variation in the SWIR and at the specific water absorption trough at 1175 nm could be related to the timing of drought stress. When drought stress occurs during the main growing period (May–July), above–ground biomass can be significantly reduced (Novotná et al. 2016) and senesced vegetation could increase due to vegetation mortality (Winkler et al. 2019). The SWIR region is efficient at identifying dry vegetation due to the presence of absorption features of dry vegetation tissues like cellulose, hemicellulose, or lignin (Asner and Lobell 2000; Daughtry 2001; Kergoat et al. 2015). Thus, our findings of lower reflectance in the SWIR region for June 2023, July 2022 and August 2023 could indicate higher senescence in that period due to lower precipitation June 2023 experienced 14% lower precipitation than June 2022. It is important to note that during July 2022 only 10.66 mm, a minimal amount of precipitation, at least 87% less than in July of 2023. As for August 2023 there was a 72% reduction in precipitation than for August of 2022. For more detailed information regarding the interannual variation in precipitation for spring and summer of 2022 and 2023, see Figure S1.

### 4.2 Biotic and Abiotic Factors Influencing Canopy Structure and Function

The timing of precipitation and the presence of C3 species are key contributors to impact canopy function as demonstrated by the conditional inference trees. In our system, C4 plant species are beginning to senesce in late summer/early fall and with sufficient rainfall some C3 and N‐fixers can regrow which is why values tend to differ interannually for August (Castillioni et al. 2020). The overall presence of *Lespedeza cuneata* (C3 species) as well as its presence later in the season influences greenness, showing that ‐*L. cuneata* can be a driver of canopy responses corroborating with the findings of our PERMANOVA on plant composition. Previous research has found that *L. cuneata* is able to alter soils to facilitate its own growth as well as increase its biomass in soils it has already colonized, can suppress native grass species, and has morphological and physiological traits consistent with drought tolerance (Allred et al. 2010; Coykendall and Houseman 2014). *Lespedeza* has also been shown to dominate canopy responses to changes in precipitation in other grassland ecosystems (Kardol et al. 2010). The inference tree also showed that plots with a higher proportion of *L. cuneata* had the highest NDVI values (Figure 3). Thus, when *L. cuneata* is present it can maintain canopy function and supersede drought effects.

The Conditional Inference Tree for LAI indicated that 2022 had higher LAI values; however, LAI reflects only canopy layers, not greenness, and includes senesced plants and litter (i.e., nonphotosynthetic vegetation) (Xu et al. 2020). Which explains why 2023 (mesic) is overall greener with higher NDVI values than 2022 (dry) but with lower LAI values. Additionally, the milder drought treatments (20% and −40%) have lower LAI values than the rest potentially due to the more severe drought treatments having less forbs and more grasses, senesced plants, and litter. Similar to the CropScan NDVI, in the LAI tree the presence of *L. cuneata* at the end of the growing season for 2022 plays important role, resulting in higher values probably due to its abundance, the species' growth form, and resiliency as canopy size and architecture determines the amount of light energy that is absorbed by the whole plant (Allred et al. 2010).

### 4.3 Plant Composition, Species Richness, Diversity & Evenness

Increases in plant diversity under severe drought treatments were driven by a decline in plant dominance (higher evenness). Castillioni et al. (2020) also found greater plant evenness in extreme drought with a reduction in plant dominance patterns. These changes in dominance patterns can also be due to drought intensity modulating community responses (Oram et al. 2023). Similar to our study, Castillioni et al. (2020) and other studies (e.g., Xu et al. 2017) did not detect changes in plant richness under drought conditions.

The high variance across treatment replicates may reflect long‐term differences in water conditions, as suggested by Castillioni et al. (2020), indicating that water availability is the primary factor shaping plant composition. Additionally, as postulated by Mariotte (2014), this low similarity could be due to differences in the subdominant plant species composition. The highest similarity was observed for the 80% reduction plots, and this similarity is due to those plots having the lowest species richness and diversity when compared to the other plots.

On average *L. cuneata* contributes 17% of the overall vegetation cover in the treatments where it is most abundant (i.e., control, addition and 60% reduction) (see Supporting Information; Figure S2). *L. cuneata* exhibits drought tolerance due to traits such as high specific leaf area (SLA), elevated leaf nitrogen, and the ability to maintain consistent daily photosynthetic activity and stomatal conductance, enabling a resource‐acquisitive strategy (Allred et al. 2010). Because of this and its overall high abundance it can impact NDVI and LAI, especially when noting that it has higher SLA than other native prairie species (Allred et al. 2010). Our only invasive, *Bothrichloa ischaemum*, was a top contributor to overall vegetation cover in under greater water availability (the water addition and 20% reduction plots). Previous research in our system has documented a shift in species dominance from 2016 to 2023 indicating an increase of *L. cuneata* (C3) and a decrease of *Schizachyrium scoparium* (C4) in the control plots (Wall et al. 2024, unpublished data).

### 4.4 Linking Canopy Function and Structure

Overall, we found that during July 2022, the driest month of that season, there was a low reflectance in the SWIR region and a negative relationship between NDVI and LAI (*N* = 9; see Figure S7) for both our severe drought and precipitation addition treatments. Coupled together, these findings showed that our C4 grasses were impacted more by the limited water than our C3 forbs and legumes. As C4 grasses tend to have higher LAI values than the forbs and legumes due to their growth form, lower LAI values during the month of July were correlated with higher greenness. Furthermore, in our system, July is when our C4 grasses are at their peak in biomass; thus, drought could have reduced biomass impacting canopy function. When water is not a limiting factor, we found a positive relationship (*R* = 0.44, *p* = 0.048; Figure S4) between CropScan NDVI and LAI during June for 2023, indicating that precipitation and the timing of it were important factors that affected canopy function.

Our conditional inference trees showed that the late season presence of *L. cuneata* increases canopy greenness and structure even as things begin to senesce in August. This finding in conjunction with the negative relationship (*R* = −0.056, *p* = 1.4e‐10; Figure S5) between LAI and NDVI and with LAI and NDWI (*R* = −0.54, *p* = 0.013) during August showed how species‐specific responses influence canopy‐level responses, with species like *L. cuneata* that have drought‐resistant traits or can maintain their biomass senesce later in the season even under water constraints.

## 5 Conclusion

Our results demonstrate that there is a higher reflectance in the visible region, associated with pigmentation (i.e., chlorophyll content), and overall higher canopy greenness in wetter years and early in the growing season; meaning annual and seasonal drought reduces canopy function. Variation in the NIR and SWIR regions reflected seasonal shifts in water availability across both years. In water limited ecosystems like grasslands, precipitation can be a dominant driver in canopy spectral reflectance responses and overall canopy function.

Our results also show divergent responses in canopy structure (LAI) and function (reflectance) where vegetation indices were consistently higher in wetter years while LAI increased under drought conditions. This decoupling between canopy structure (LAI) and function (reflectance) suggests that drought could be increasing the amount of nonphotosynthetic vegetation with increased senesced material (litter). However, we did not measure litter throughout the growing season; thus, this potential mechanism remains an inference and, as such, further research is needed to understand this seasonal and climatically dependent decoupling.

Precipitation also mediated shifts in plant dominance—from C4 dominant to C3 dominant—and evenness, and under drought events (experimental and temporal variation) there were significant impacts to canopy structure and function. Those impacts or changes can be closely tied to both the presence and shifts in the abundance of *Lespedeza cuneata* , a dominant C3 species within our site, suggesting that its drought resistant traits (Allred et al. 2010; Coykendall and Houseman 2014) influence canopy function (greenness and water content) and structure (LAI). Plants at different phenological stages are thought to vary in their resistance and resilience to drought, and as such, ecosystem responses to drought may vary with different seasonal drought timing (Li et al. 2022). Previous studies report that early‐season droughts substantially reduce current‐year biomass by limiting peak biomass accumulation (D'Orangeville et al. 2018; Meng et al. 2019; Li et al. 2022). In contrast, late season droughts have been found to have little adverse impacts on current‐year biomass but large negative legacy effects on the following year biomass (Kannenberg et al. 2019; Li et al. 2022). Thus, drought effects on ecosystems can be context‐dependent (Li et al. 2022). In our case, experimental drought contributed towards a shift in plant composition and reductions in C4 grass dominance. These context‐dependent responses highlight the need for future work linking leaf‐level traits and canopy architecture to spectral signatures, particularly to better understand how drought‐driven changes in community composition feedback to ecosystem function and remote‐sensing assessments.

## Author Contributions

**Mariela Encarnación Ojeda:** conceptualization (lead), data curation (lead), formal analysis (equal), funding acquisition (equal), investigation (lead), methodology (lead), project administration (lead), writing – original draft (lead), writing – review and editing (lead). **Xiangming Xiao:** data curation (supporting), formal analysis (supporting), methodology (equal), visualization (supporting), writing – review and editing (supporting). **Emily U. Nguyen:** data curation (supporting), investigation (supporting). **Lara Souza:** conceptualization (equal), data curation (equal), formal analysis (equal), funding acquisition (equal), investigation (equal), methodology (equal), project administration (equal), supervision (equal), writing – original draft (equal), writing – review and editing (equal).

## Funding

This work was supported by the Office of Experimental Program to Stimulate Competitive Research, OIA‐1301789.

## Disclosure

Statement on inclusion: This study brings together local scientists and cites literature published by scientists from the region and other areas.

## Conflicts of Interest

The authors declare no conflicts of interest.

## Supporting information


**Table S1:** PERMANOVA pairwise results showing block differences across years with Bonferroni correction for adjusted *p*‐values.
**Table S2:** Post hoc Tukey HSD showing treatment differences in the AUC for the NIR region (*N* = 9).
**Table S3:** Pairwise comparisons of treatment effects on Evenness (back‐transformed estimates) and Diversity using Tukey's adjustment for multiple comparisons. Estimates, standard errors (SE), degrees of freedom (df), *t*‐ratios, and adjusted *p*‐values are reported for each group.
**Table S4:** Similarity Percentages (SIMPER) test showing the average dissimilarity between treatments (*N* = 21).
**Figure S1:** 1–month SPI for Spring and Summer for 2022 and 2023. Line at center is normality and values above 1 or −1 indicate values above average precipitation or below average precipitation.
**Figure S2:** Relativized *Lespedeza cuneata* Cover Bar Plots for 2022 and 2023. *Lespedeza* is highlighted in red and increases later in the season for both years.
**Figure S3:** Canopy reflectance curves for the 2022 and 2023 summer growing seasons. 2022 above and 2023 below.
**Figure S4:** (A) Spearman correlations between LAI and CropScan NDVI for all plots (*N* = 21) showing both seasonal and interannual variation. (B) Spearman correlations between LAI and NDVI for all plots (*N* = 9) showing both seasonal and interannual variation. (C) Spearman correlations between LAI and NDWI for all plots (*N* = 9) showing both seasonal and interannual variation.
**Figure S5:** (A) Spearman correlations between LAI and CropScan NDVI for all plots (*N* = 21) showing seasonal variation. (B) Spearman correlations between LAI and NDVI for the extreme plots (*N* = 9). (C) Spearman correlations between LAI and NDWI for the extreme plots (*N* = 9).
**Figure S6:** (A) Spearman correlations between LAI and CropScan NDVI for all plots (*N* = 21). (B) Spearman correlations between LAI and NDVI for the extreme plots (*N* = 9). (C) Spearman correlations between LAI and NDWI for the extreme plots (*N* = 9).
**Figure S7:** (A) Spearman correlations between LAI and CropScan NDVI interannually across all plots (*N* = 21). (B) Spearman correlations between LAI and NDVI interannually across precipitation gradient extremes (*N* = 9). (C) Spearman correlations between LAI and NDWI interannually across precipitation gradient extremes (*N* = 9).
**Figure S8:** (A) Spearman correlations between LAI and CropScan NDVI showing seasonal variation across the precipitation gradient extremes (*N* = 21). (B) Spearman correlations between LAI and NDVI showing seasonal variation across the precipitation gradient extremes (*N* = 9). (C) Spearman correlations between LAI and NDWI showing seasonal variation across the precipitation gradient extremes (*N* = 9).

## Data Availability

Data will be available at the Dryad Digital Repository under the DOI: 10.5061/dryad.280gb5n3b.
