# Supplementary material for: Linking Grassland Canopy Structure and Function Responses to Field Experimental Drought Both Seasonally and Interannually
Source: Ecol Evol. 2026 Apr 16;16(4):e73447. doi: 10.1002/ece3.73447 (PMC13087442; doi:10.1002/ece3.73447)
Supplement: Supplementary file 1 — Table S1: PERMANOVA pairwise results showing block differences across years with Bonferroni correction for adjusted p‐values. Table S2: Post hoc Tukey HSD showing treatment differences in the AUC for the NIR region (N = 9). Table S3: Pairwise comparisons of treatment effects on Evenness (back‐transformed estimates) and Diversity using Tukey's adjustment for multiple comparisons. Estimates, standard errors (SE), degrees of freedom (df), t‐ratios, and adjusted p‐values are reported for each group. Table S4: Similarity Percentages (SIMPER) test showing the average dissimilarity between treatments (N = 21). Figure S1: 1–month SPI for Spring and Summer for 2022 and 2023. Line at center is normality and values above 1 or −1 indicate values above average precipitation or below average precipitation. Figure S2: Relativized Lespedeza cuneata Cover Bar Plots for 2022 and 2023. Lespedeza is highlighted in red and increases later in the season for both years. Figure S3: Canopy reflectance curves for the 2022 and 2023 summer growing seasons. 2022 above and 2023 below. Figure S4: (A) Spearman correlations between LAI and CropScan NDVI for all plots (N = 21) showing both seasonal and interannual variation. (B) Spearman correlations between LAI and NDVI for all plots (N = 9) showing both seasonal and interannual variation. (C) Spearman correlations between LAI and NDWI for all plots (N = 9) showing both seasonal and interannual variation. Figure S5: (A) Spearman correlations between LAI and CropScan NDVI for all plots (N = 21) showing seasonal variation. (B) Spearman correlations between LAI and NDVI for the extreme plots (N = 9). (C) Spearman correlations between LAI and NDWI for the extreme plots (N = 9). Figure S6: (A) Spearman correlations between LAI and CropScan NDVI for all plots (N = 21). (B) Spearman correlations between LAI and NDVI for the extreme plots (N = 9). (C) Spearman correlations between LAI and NDWI for the extreme plots (N = 9). Figure S7: (A) Spearman correlat [file ECE3-16-e73447-s001.docx]

**Supplementary Information/Appendix**

*Abiotic Factors: Precipitation and Soil Moisture*

We collected soil moisture (%) for the precipitation gradient extremes (0%, +50% and -100%) at 10 and 20 cm in depth using 12 cm Water Content Reflectometers (Campbell Scientific, <https://www.campbellsci.com/cs655-lc>). The sensor collected data at 5–minute intervals; for the purposes of this study, only the monthly average for the summer growing seasons of 2022 and 2023 was used. An ANOVA was conducted to assess if soil moisture at both 4 and 8 inches of depth varied across treatment (fixed effect), month, year, or its interactions (random effects). An ANOVA was also conducted to assess if 1-month SPI varied across months, year and even season (spring or summer). The 1-month SPI indicates that the month of July experienced moderately dry conditions in 2022 and experienced moderately wet conditions in 2023. Additionally, August 2023 experienced moderately dry conditions. The ANOVA for soil moisture showed that at 10 cm it exhibits month (p=1.69e-05) and year (p=4.64e-09) differences and at 20 cm soil moisture exhibits treatment (p=4.61e-04) and year (p=1.53e-07) differences as well as their interaction (p=0.002). At both depths, soil moisture was higher for 2023 rather than 2022. Soil moisture at 10 cm was higher in 2023 than 2022 and August was the driest month. Soil moisture at 20 cm was higher in 2023 than in 2022. We found that the control plots were 27% wetter in 2023 than 2022, while the drought and addition plots were 62% and 68% wetter than in the previous year. Overall, the addition had 33% higher soil moisture at 20 cm of depth than the control and drought plots regardless of year.

**Table S1** - PERMANOVA pairwise results showing block differences across years with Bonferroni correction for adjusted p-values.

| PERMANOVA Pairwise Table | | | |
| --- | --- | --- | --- |
| Groups | t | p-values | Block |
| 2022, 2023 | 2.1851 | 0.001* | 1 |
| 2022, 2023 | 1.1254 | 0.2837 | 2 |
| 2022, 2023 | 2.342 | 0.0003* | 3 |

**Table S2** – Post-hoc Tukey HSD showing treatment differences in the AUC for the NIR region (N=9).

| NIR AUC Tukey HSD | | |
| --- | --- | --- |
| Groups | estimate | p-values |
| 0%, -100% | -6.27 | 0.0848 |
| -100%, 50% | 28.2 | 0.0444* |
| 0%, 50% | 34.5 | 8.63e-03* |

**Table S3** – Pairwise comparisons of treatment effects on Evenness (back-transformed estimates) and Diversity using Tukey’s adjustment for multiple comparisons. Estimates, standard errors (SE), degrees of freedom (df), t-ratios, and adjusted p-values are reported for each group.

| **Response** | **Groups** | **Estimate** | **SE** | **df** | **t-ratio** | **p-value** |
| --- | --- | --- | --- | --- | --- | --- |
| Evenness | 0%, 50% | 0.13056 | 0.0423 | 77 | 3.083 | 0.0432* |
|  | 0%, -100% | -0.10782 | 0.0502 | 77 | -2.146 | 0.337 |
|  | 0%, -80% | 0.07715 | 0.044 | 77 | 1.754 | 0.5826 |
|  | 0%, -60% | -0.06045 | 0.0486 | 77 | -1.245 | 0.8742 |
|  | 0%, -40% | -0.05119 | 0.0482 | 77 | -1.061 | 0.9374 |
|  | 0%, -20% | -0.03856 | 0.0478 | 77 | -0.807 | 0.9836 |
|  | 50%, -100% | -0.23838 | 0.0464 | 77 | -5.135 | <0.0001* |
|  | 50%, -80% | -0.05341 | 0.0396 | 77 | -1.35 | 0.8261 |
|  | 50%, -60% | -0.19101 | 0.0446 | 77 | -4.282 | 0.001* |
|  | 50%, -40% | -0.18175 | 0.0443 | 77 | -4.107 | 0.0018* |
|  | 50%, -20% | -0.16912 | 0.0438 | 77 | -3.863 | 0.0042* |
|  | -100%, -80% | 0.18497 | 0.0479 | 77 | 3.859 | 0.0042* |
|  | -100%, -60% | 0.04737 | 0.0522 | 77 | 0.908 | 0.9702 |
|  | -100%, -40% | 0.05663 | 0.0519 | 77 | 1.092 | 0.9286 |
|  | -100%, -20% | 0.06926 | 0.0515 | 77 | 1.346 | 0.8279 |
|  | -80%, -60% | -0.1376 | 0.0462 | 77 | -2.98 | 0.0565 |
|  | -80%, -40% | -0.12834 | 0.0458 | 77 | -2.8 | 0.0886 |
|  | -80%, -20% | -0.11571 | 0.0454 | 77 | -2.55 | 0.1563 |
|  | -60%, -40% | 0.00926 | 0.0502 | 77 | 0.184 | 1 |
|  | -60%, -20% | 0.02189 | 0.0498 | 77 | 0.439 | 0.9994 |
|  | -40%, -20% | 0.01263 | 0.0495 | 77 | 0.255 | 1 |
| Diversity | 0%, 50% | 0.428 | 0.0903 | 42 | 4.738 | 0.0005* |
|  | 0%, -100% | -0.1392 | 0.0903 | 42 | -1.541 | 0.7191 |
|  | 0%, -80% | 0.2446 | 0.0903 | 42 | 2.707 | 0.1216 |
|  | 0%, -60% | 0.0357 | 0.0903 | 42 | 0.395 | 0.9997 |
|  | 0%, -40% | -0.1938 | 0.0903 | 42 | -2.145 | 0.3465 |
|  | 0%, -20% | -0.081 | 0.0903 | 42 | -0.897 | 0.971 |
|  | 50%, -100% | -0.5672 | 0.0903 | 42 | -6.279 | <0.0001* |
|  | 50%, -80% | -0.1835 | 0.0903 | 42 | -2.031 | 0.4115 |
|  | 50%, -60% | -0.3923 | 0.0903 | 42 | -4.343 | 0.0016* |
|  | 50%, -40% | -0.6218 | 0.0903 | 42 | -6.883 | <0.0001* |
|  | 50%, -20% | -0.5091 | 0.0903 | 42 | -5.635 | <0.0001* |
|  | -100%, -80% | 0.3838 | 0.0903 | 42 | 4.248 | 0.0021* |
|  | -100%, -60% | 0.1749 | 0.0903 | 42 | 1.936 | 0.4692 |
|  | -100%, -40% | -0.0546 | 0.0903 | 42 | -0.604 | 0.9963 |
|  | -100%, -20% | 0.0582 | 0.0903 | 42 | 0.644 | 0.9948 |
|  | -80%, -60% | -0.2089 | 0.0903 | 42 | -2.312 | 0.2624 |
|  | -80%, -40% | -0.4384 | 0.0903 | 42 | -4.852 | 0.0003* |
|  | -80%, -20% | -0.3256 | 0.0903 | 42 | -3.604 | 0.0134* |
|  | -60%, -40% | -0.2295 | 0.0903 | 42 | -2.54 | 0.1714 |
|  | -60%, -20% | -0.1167 | 0.0903 | 42 | -1.292 | 0.8517 |
|  | -40%, -20% | 0.428 | 0.0903 | 42 | 4.738 | 0.0005* |

**Table S4** – Similarity Percentages (SIMPER) test showing the average dissimilarity between treatments (N=21).

| Groups | Species | Contribution (%) | Average Dissimilarity (%) |
| --- | --- | --- | --- |
| 50%, -20% | *Bothrichloa ischaemum* | 20.33 | 76.99 |
|  | *Lespedeza cuneata* | 13.34 |  |
|  | *Sporobolus compositus* | 9.99 |  |
|  | *Symphyotrichum ericoides* | 8.32 |  |
|  | *Schizachyrium scoparium* | 7.37 |  |
| 50%, -40% | *Bothrichloa ischaemum* | 16.5 | 81.78 |
|  | *Lespedeza cuneata* | 12.55 |  |
|  | *Schizachyrium scoparium* | 11.34 |  |
|  | *Sporobolus compositus* | 9.42 |  |
|  | *Croton monanthogynus* | 8.86 |  |
| 20%, -40% | *Schizachyrium scoparium* | 11.8 | 69.2 |
|  | *Bothrichloa ischaemum* | 10.75 |  |
|  | *Stenaria nigricans* | 9.43 |  |
|  | *Croton monanthogynus* | 9.4 |  |
|  | *Symphyotrichum ericoides* | 9.3 |  |
| 50%, -60% | *Bothrichloa ischaemum* | 18.2 | 74.06 |
|  | *Lespedeza cuneata* | 16.4 |  |
|  | *Sporobolus compositus* | 11.03 |  |
|  | *Symphyotrichum ericoides* | 10.36 |  |
|  | *Sorghastrum nutans* | 10.31 |  |
| 20%, -60% | *Schizachyrium scoparium* | 10.63 | 72.74 |
|  | *Bothrichloa ischaemum* | 10.31 |  |
|  | *Sorghastrum nutans* | 10.16 |  |
|  | *Sporobolus compositus* | 9.51 |  |
|  | *Symphyotrichum ericoides* | 9.5 |  |
| 40%, -60% | *Schizachyrium scoparium* | 14.09 | 76.51 |
|  | *Sorghastrum nutans* | 9.82 |  |
|  | *Symphyotrichum ericoides* | 9.79 |  |
|  | *Sporobolus compositus* | 9.02 |  |
|  | *Croton monanthogynus* | 8.9 |  |
| 50%, 0% | *Schizachyrium scoparium* | 20.17 | 72.42 |
|  | *Sorghastrum nutans* | 18.04 |  |
|  | *Symphyotrichum ericoides* | 12.55 |  |
|  | *Sporobolus compositus* | 11.17 |  |
|  | *Croton monanthogynus* | 8.72 |  |
| 20%, 0% | *Lespedeza cuneata* | 16.02 | 73.56 |
|  | *Bothrichloa ischaemum* | 10.6 |  |
|  | *Sorghastrum nutans* | 10.17 |  |
|  | *Symphyotrichum ericoides* | 9.39 |  |
|  | *Sporobolus compositus* | 8.18 |  |
|  | *Stenaria nigricans* | 7.14 |  |
| 40%, 0% | *Lespedeza cuneata* | 15.07 | 78.19 |
|  | *Sorghastrum nutans* | 11.4 |  |
|  | *Schizachyrium scoparium* | 11.15 |  |
|  | *Croton monanthogynus* | 8.54 |  |
|  | *Symphyotrichum ericoides* | 8.49 |  |
| 60%, 0% | *Lespedeza cuneata* | 18.09 | 61.57 |
|  | *Sorghastrum nutans* | 14.58 |  |
|  | *Symphyotrichum ericoides* | 12.92 |  |
|  | *Sporobolus compositus* | 11.74 |  |
|  | *Schizachyrium scoparium* | 9.34 |  |

**Figure S1.** 1–month SPI for Spring and Summer for 2022 and 2023. Line at center is normality and values above 1 or -1 indicate values above average precipitation or below average precipitation.


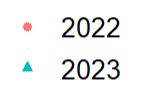

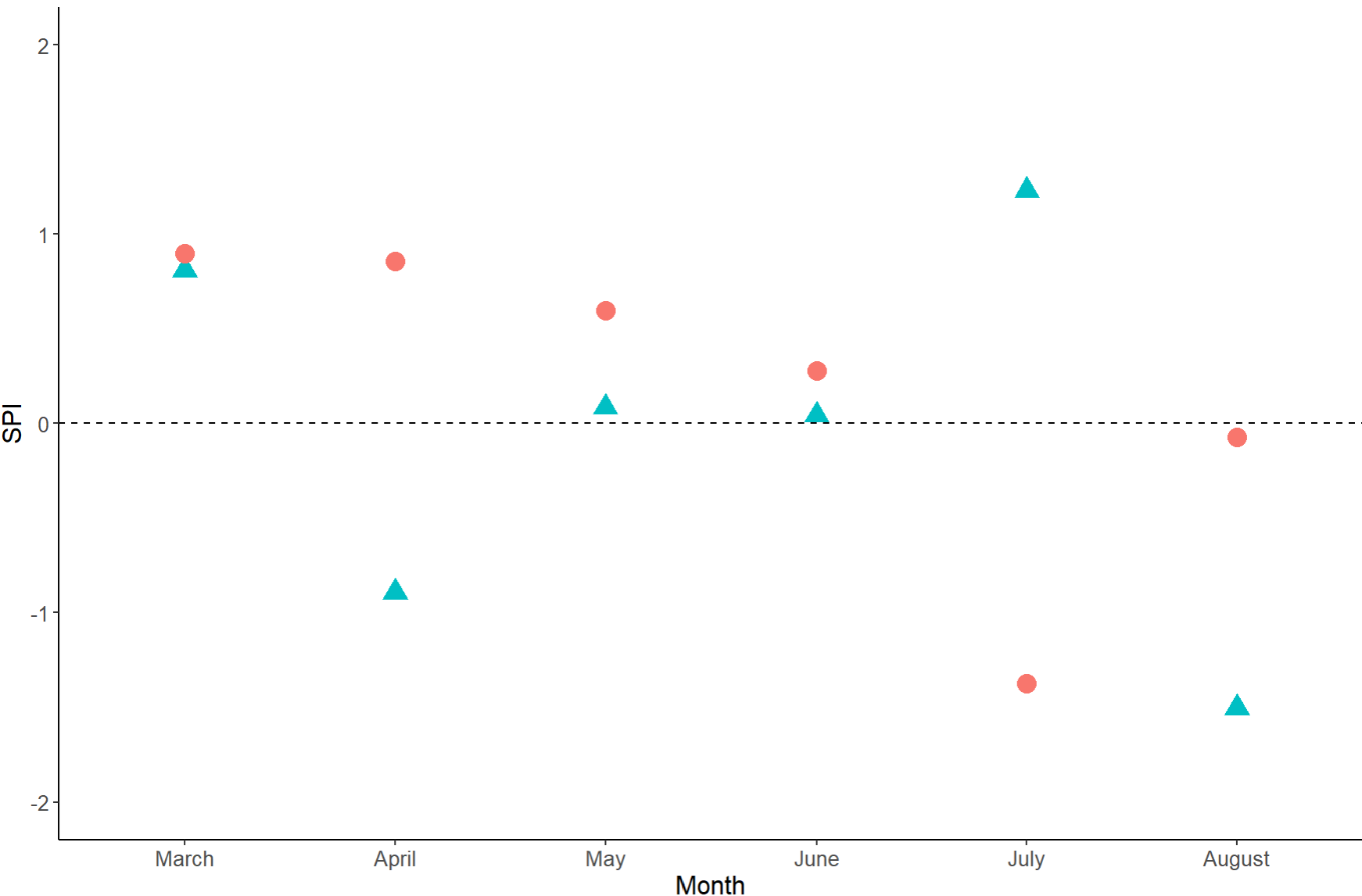


**Figure S2.** Relativized *Lespedeza cuneata* Cover Bar Plots for 2022 and 2023. *Lespedeza* is highlighted in red and increases later in the season for both years.


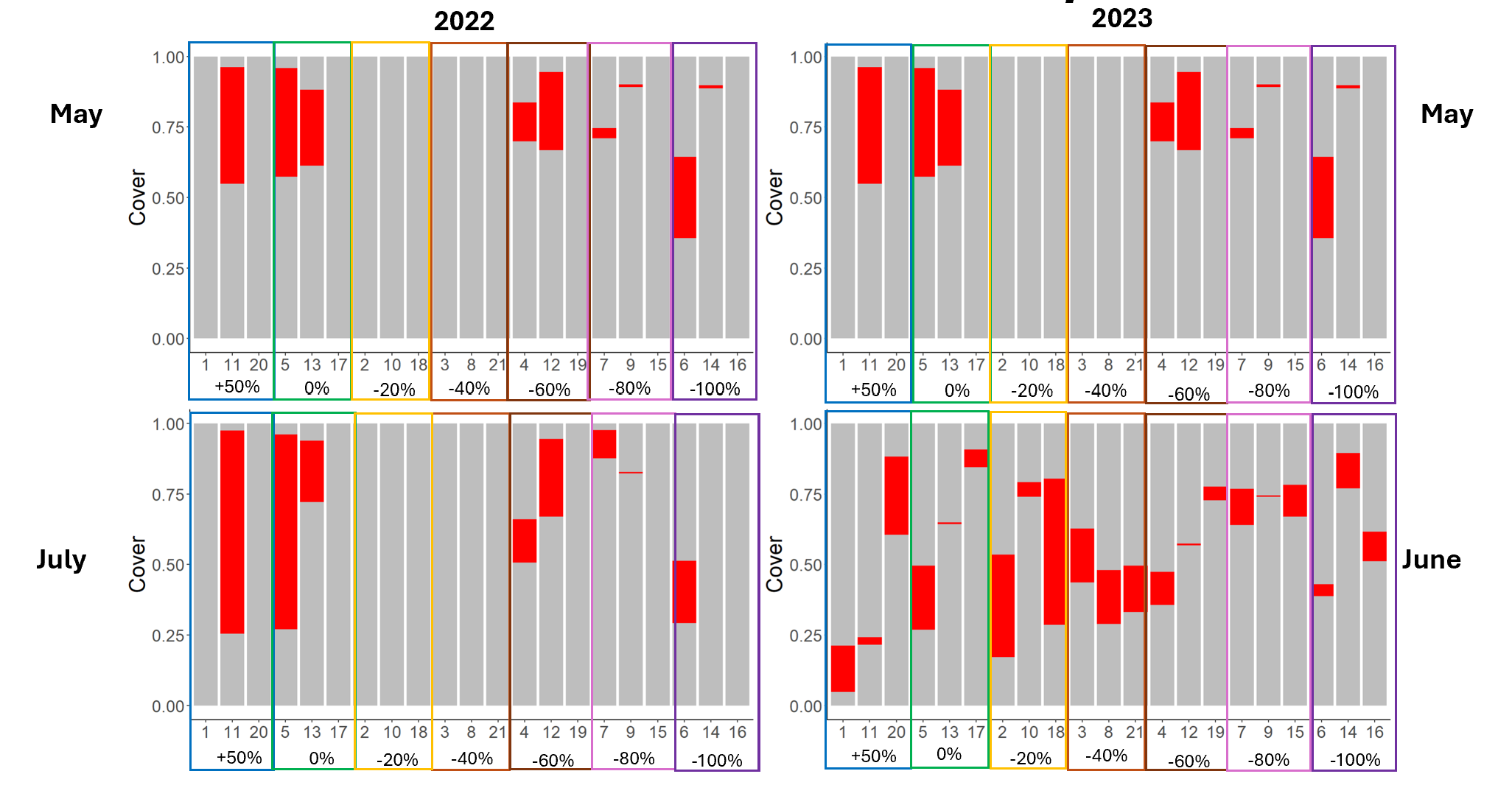


**Figure S3.** Canopy reflectance curves for the 2022 and 2023 summer growing seasons. 2022 above and 2023 below.


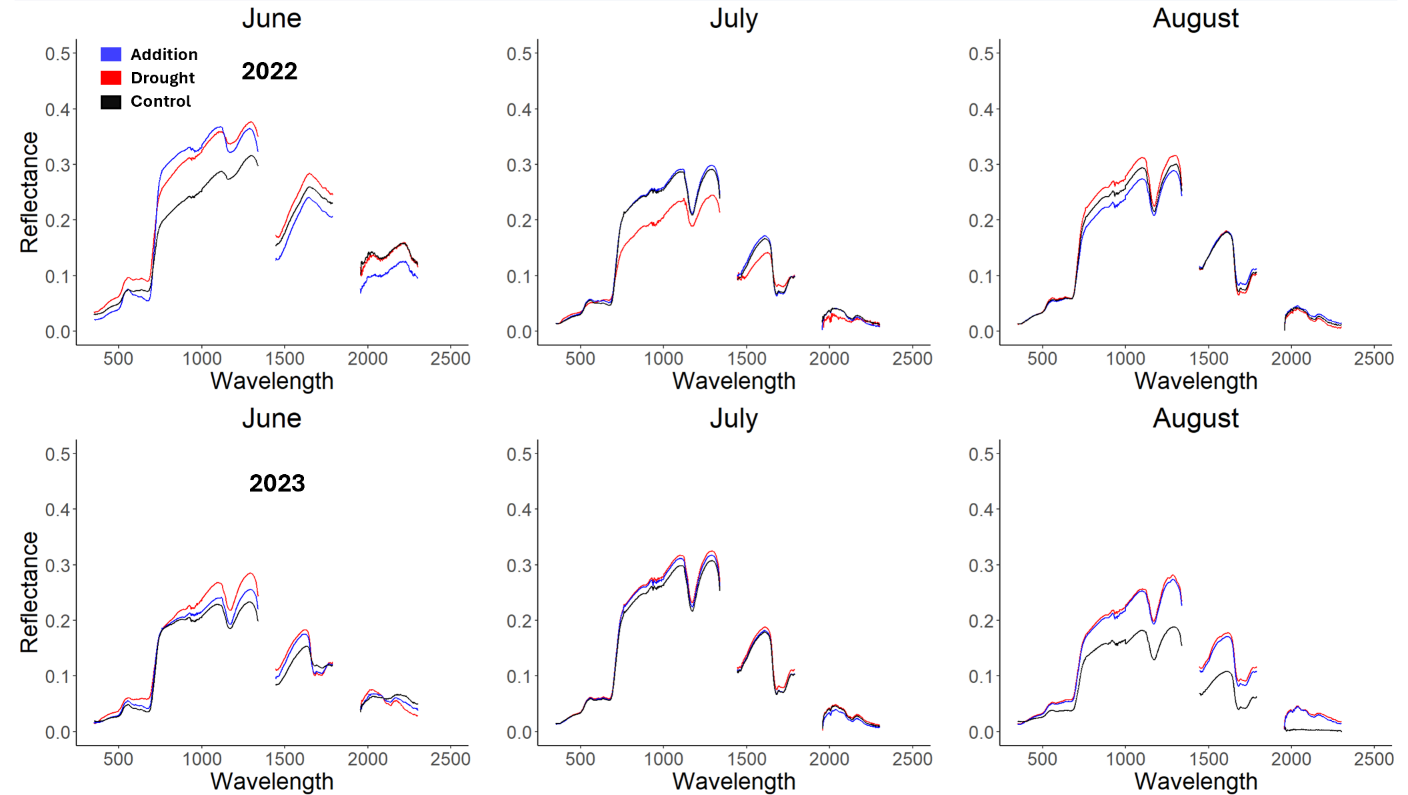
**Figure S4.** A) Spearman correlations between LAI and CropScan NDVI for all plots (N=21) showing both seasonal and interannual variation. B) Spearman correlations between LAI and NDVI for all plots (N=9) showing both seasonal and interannual variation. C) Spearman correlations between LAI and NDWI for all plots (N=9) showing both seasonal and interannual variation.

(A)

**
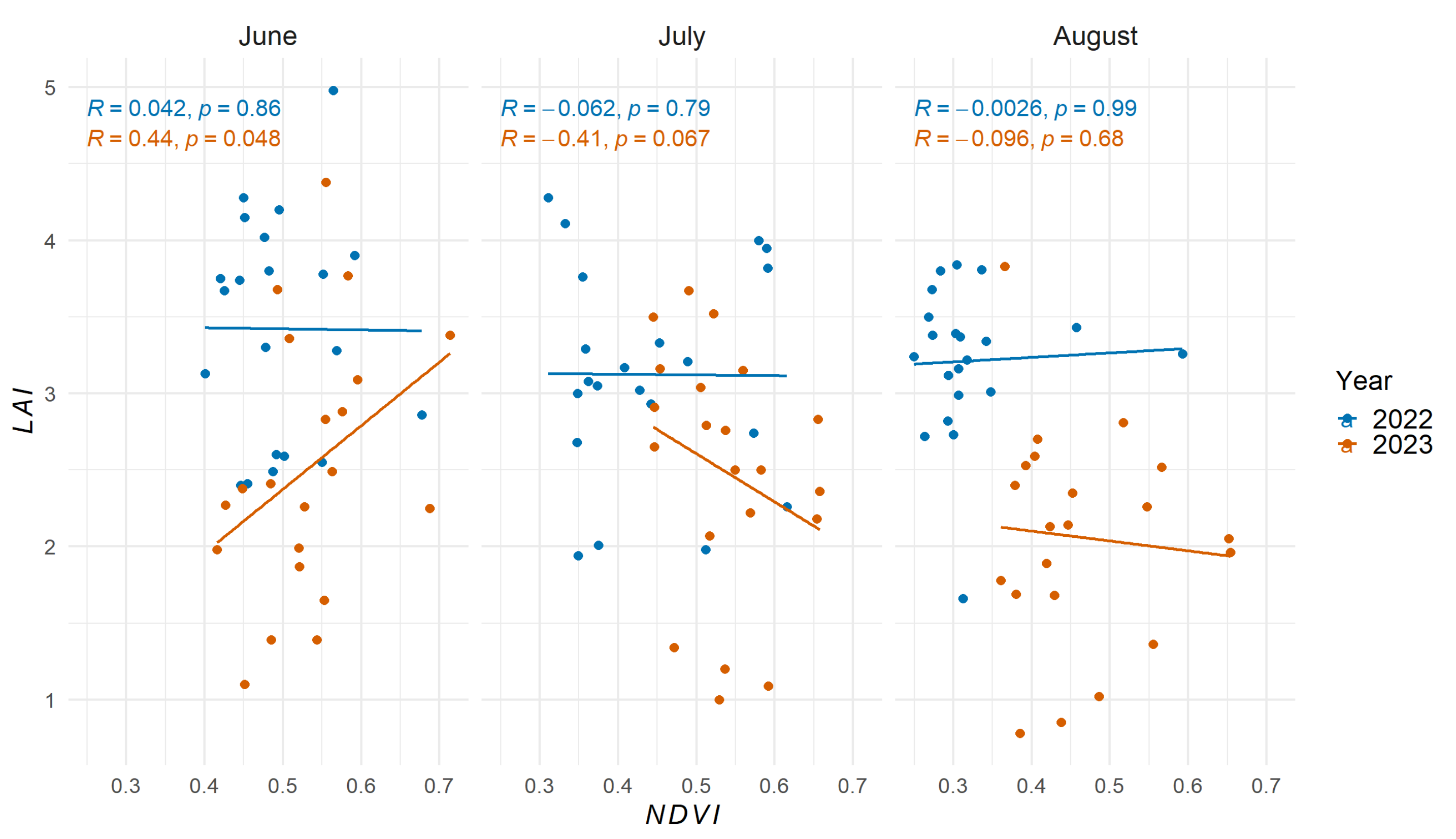
**

(B)


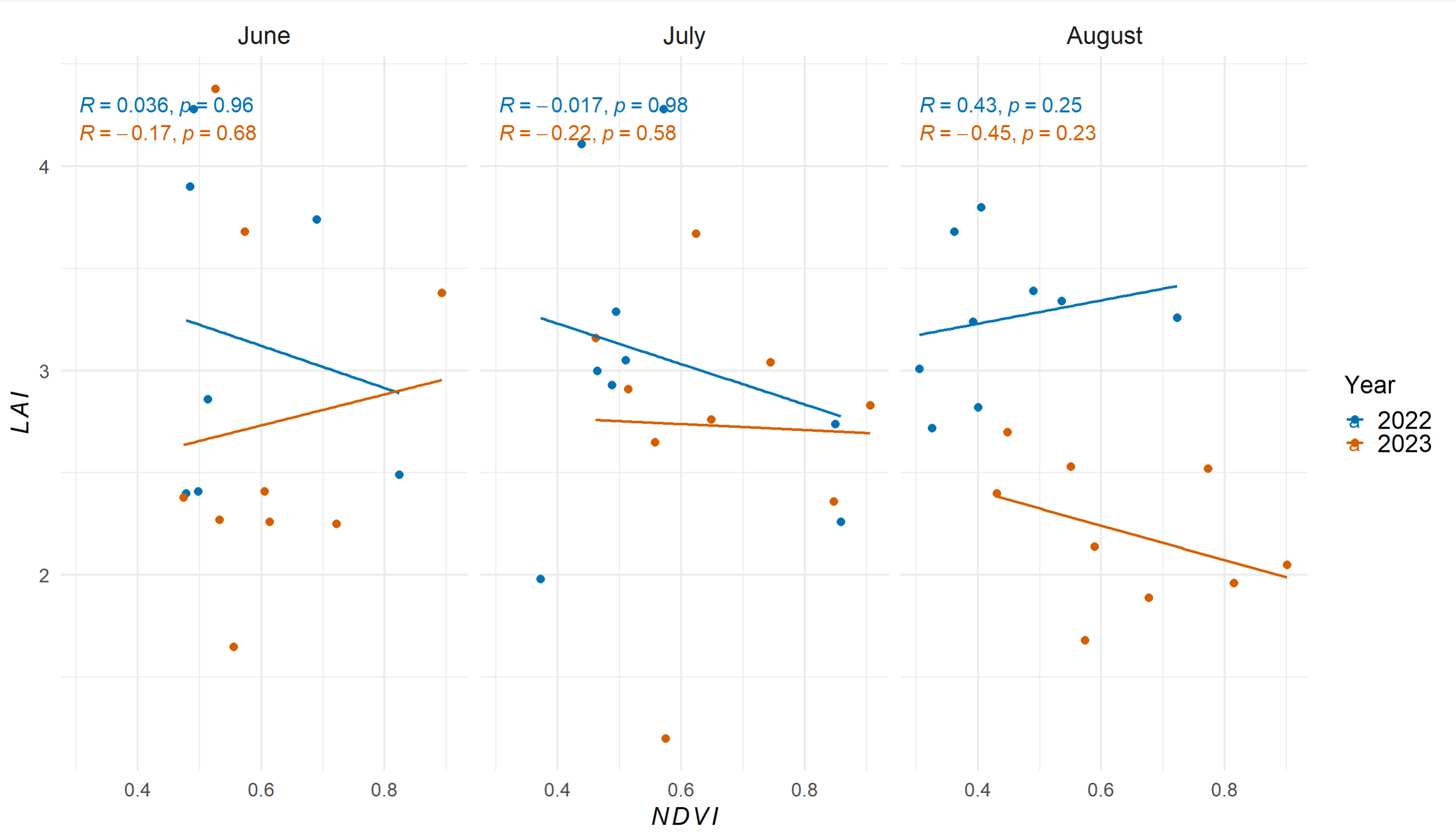


(C)


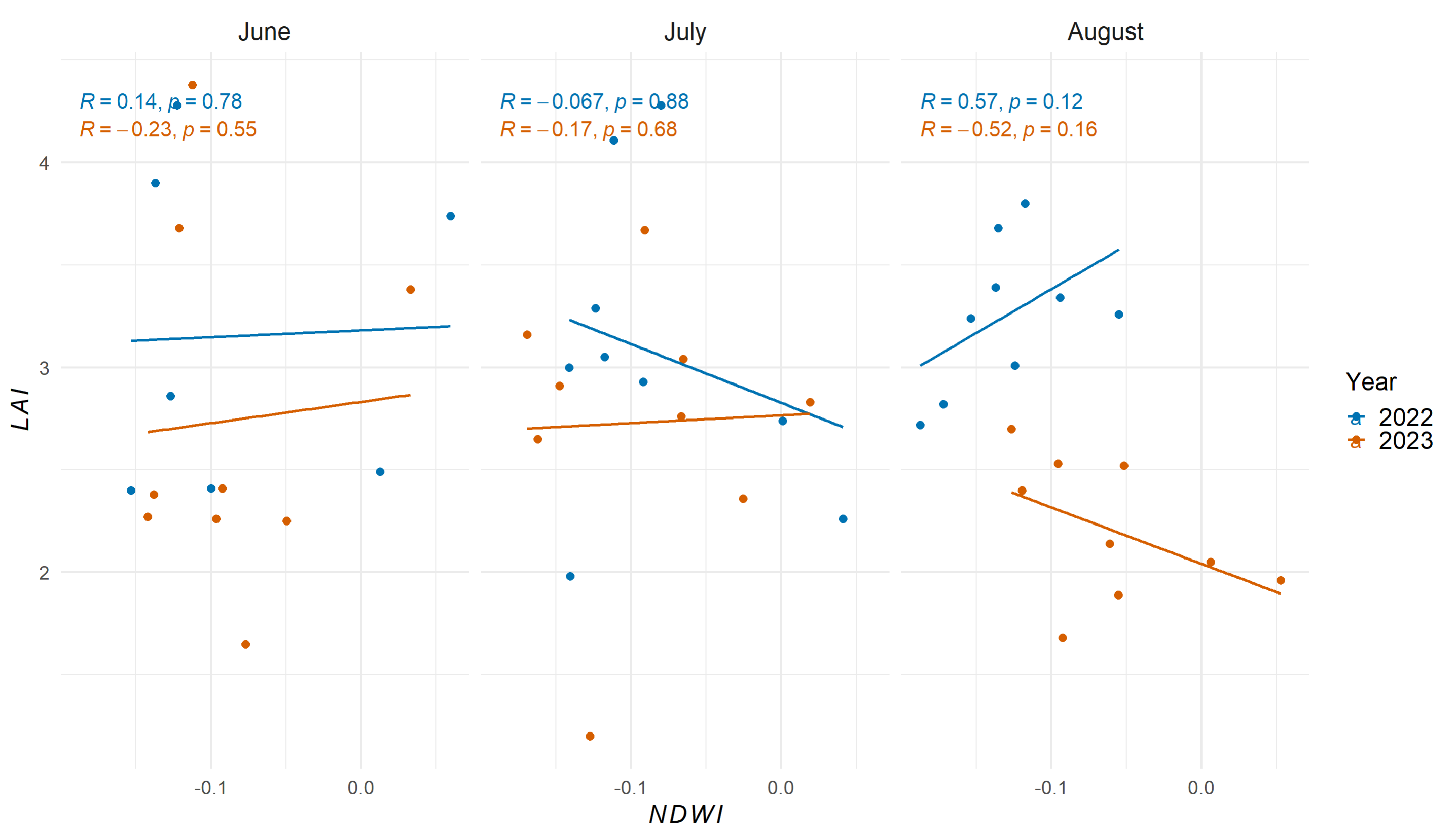


**Figure S5.** A) Spearman correlations between LAI and CropScan NDVI for all plots (N=21) showing seasonal variation. B) Spearman correlations between LAI and NDVI for the extreme plots (N=9). C) Spearman correlations between LAI and NDWI for the extreme plots (N=9).

(A)

**
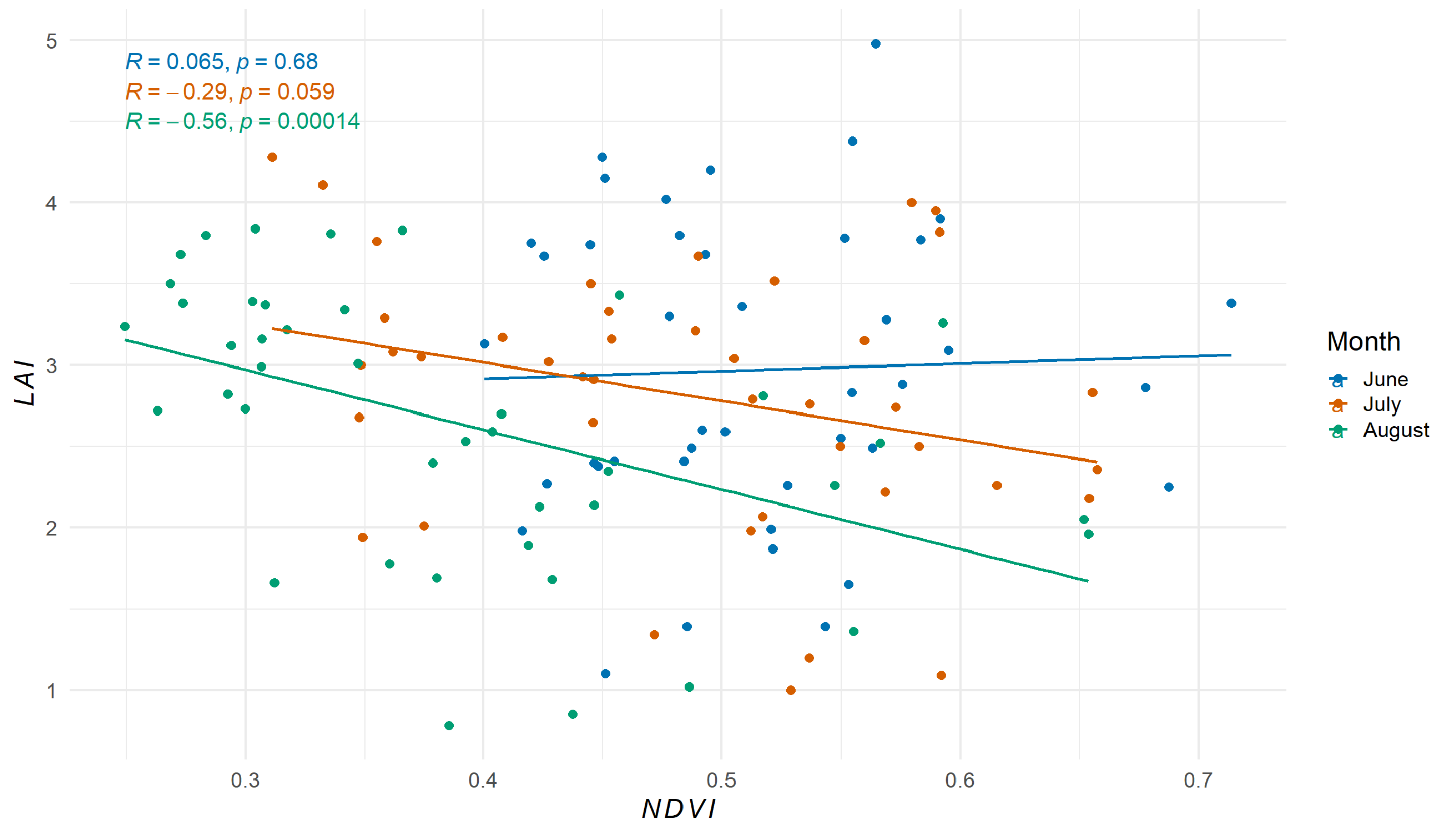
**

(B)

**
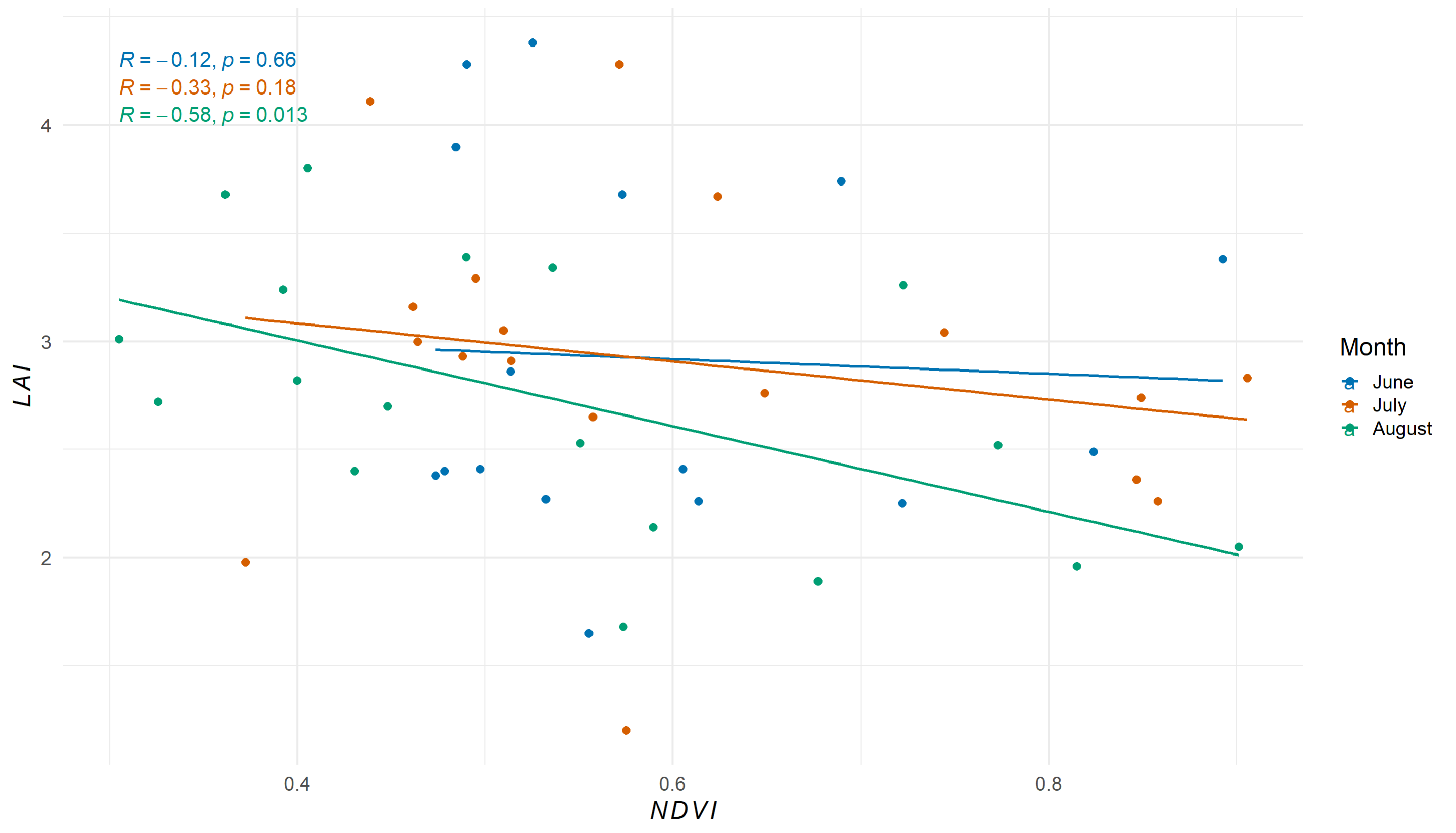
**

(C)

**
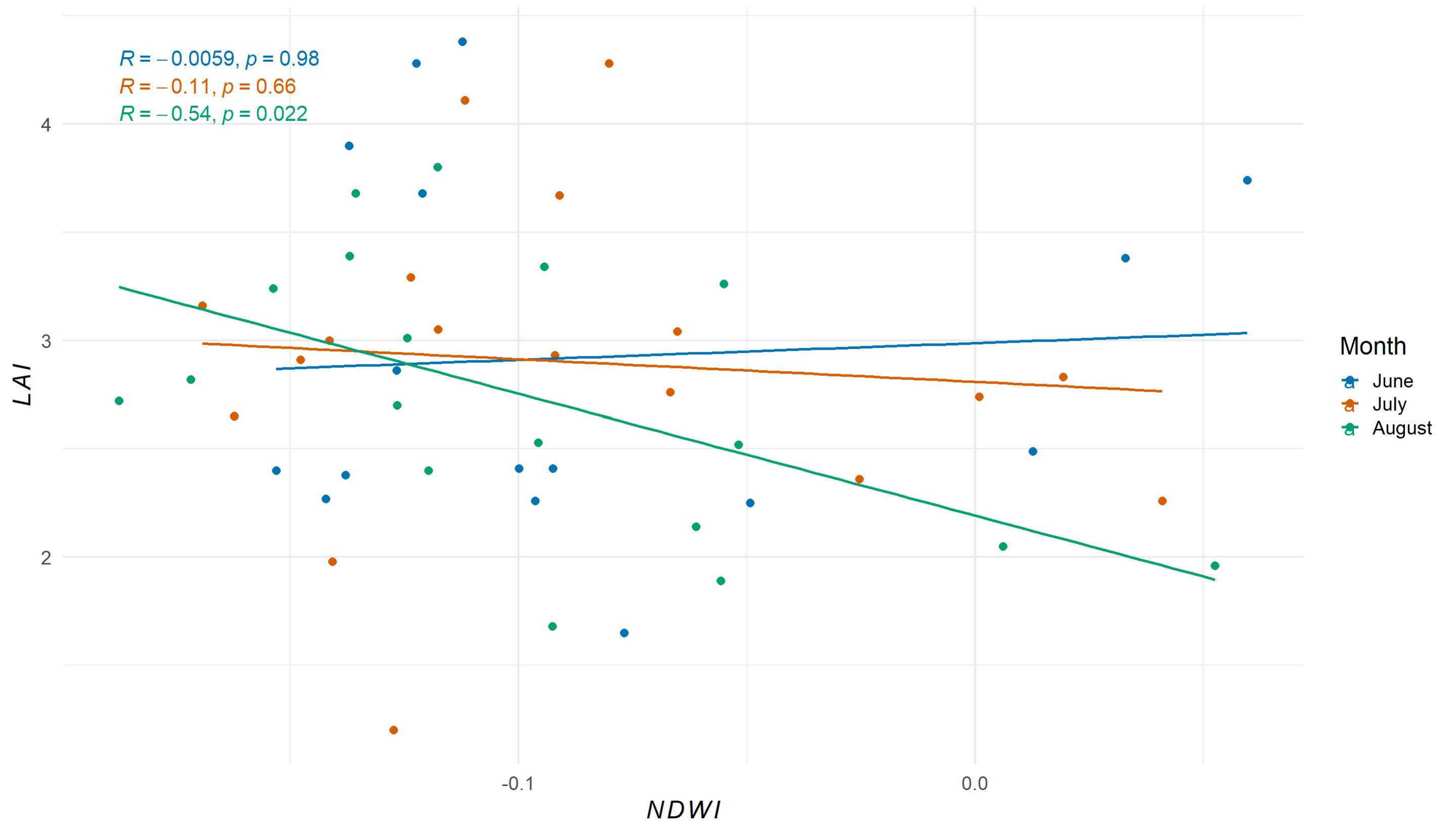
**

**Figure S6.**A) Spearman correlations between LAI and CropScan NDVI for all plots (N=21). B) Spearman correlations between LAI and NDVI for the extreme plots (N=9).  C) Spearman correlations between LAI and NDWI for the extreme plots (N=9).

(A)


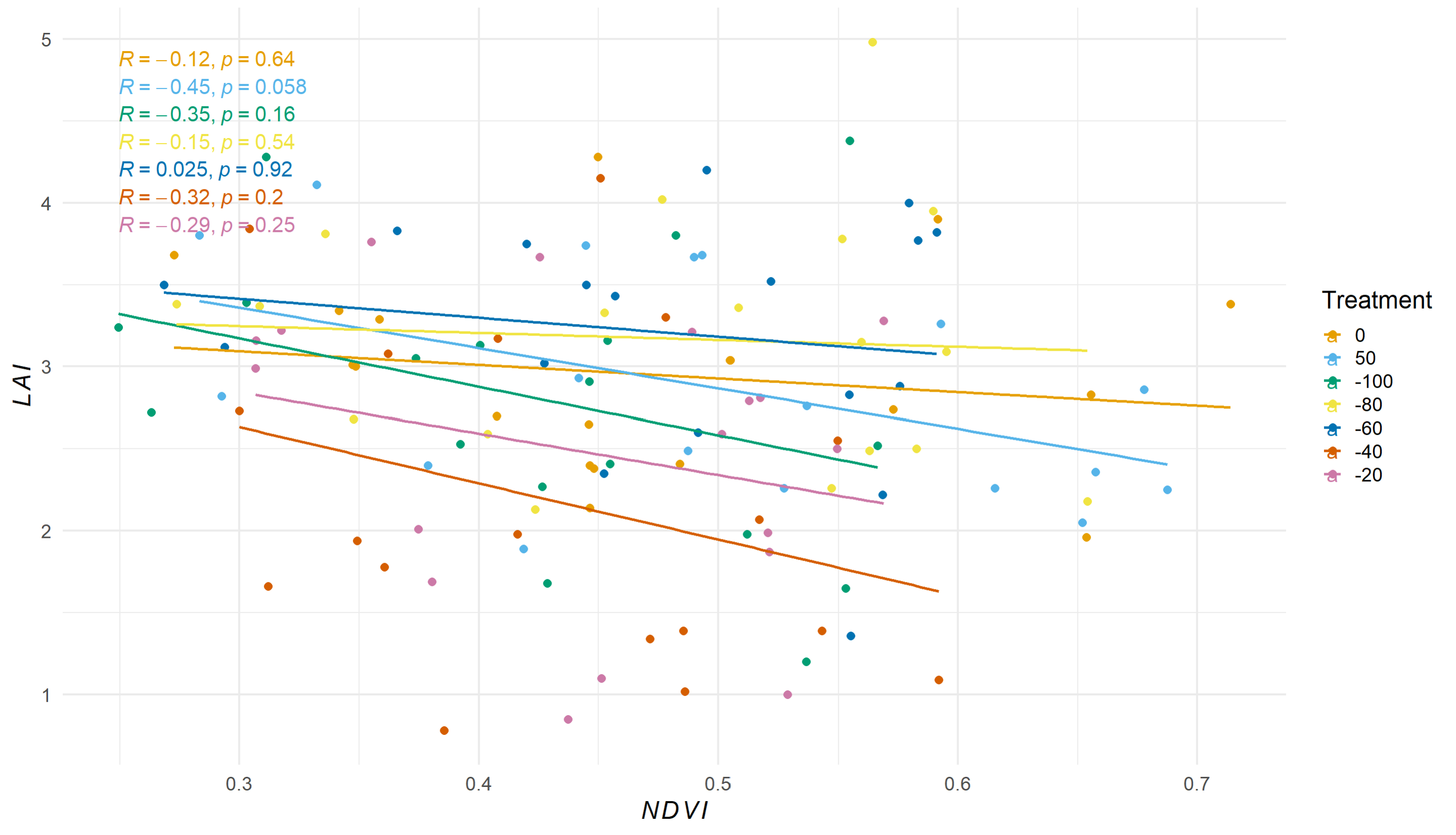


(B)


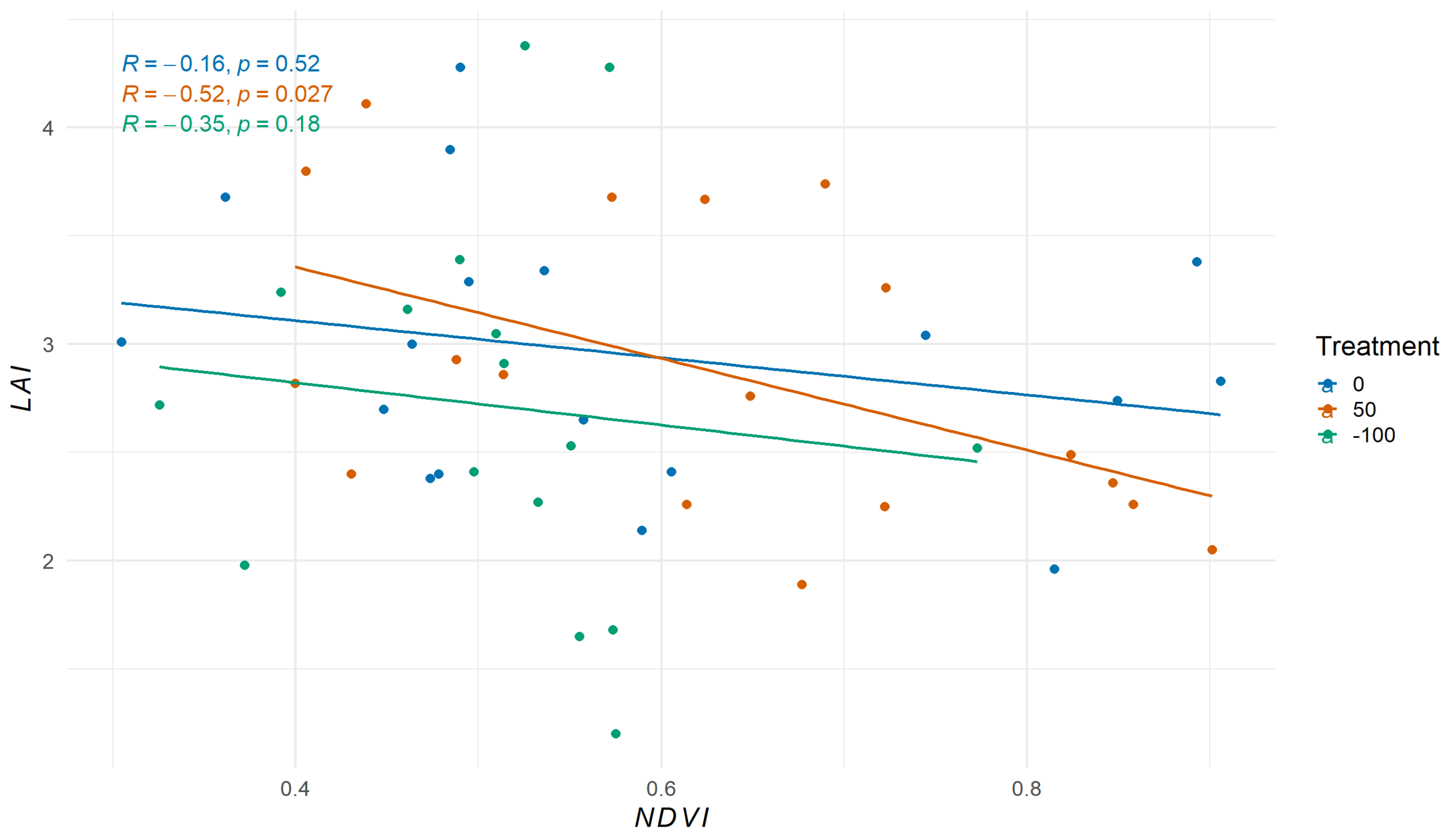


**(C)**

**
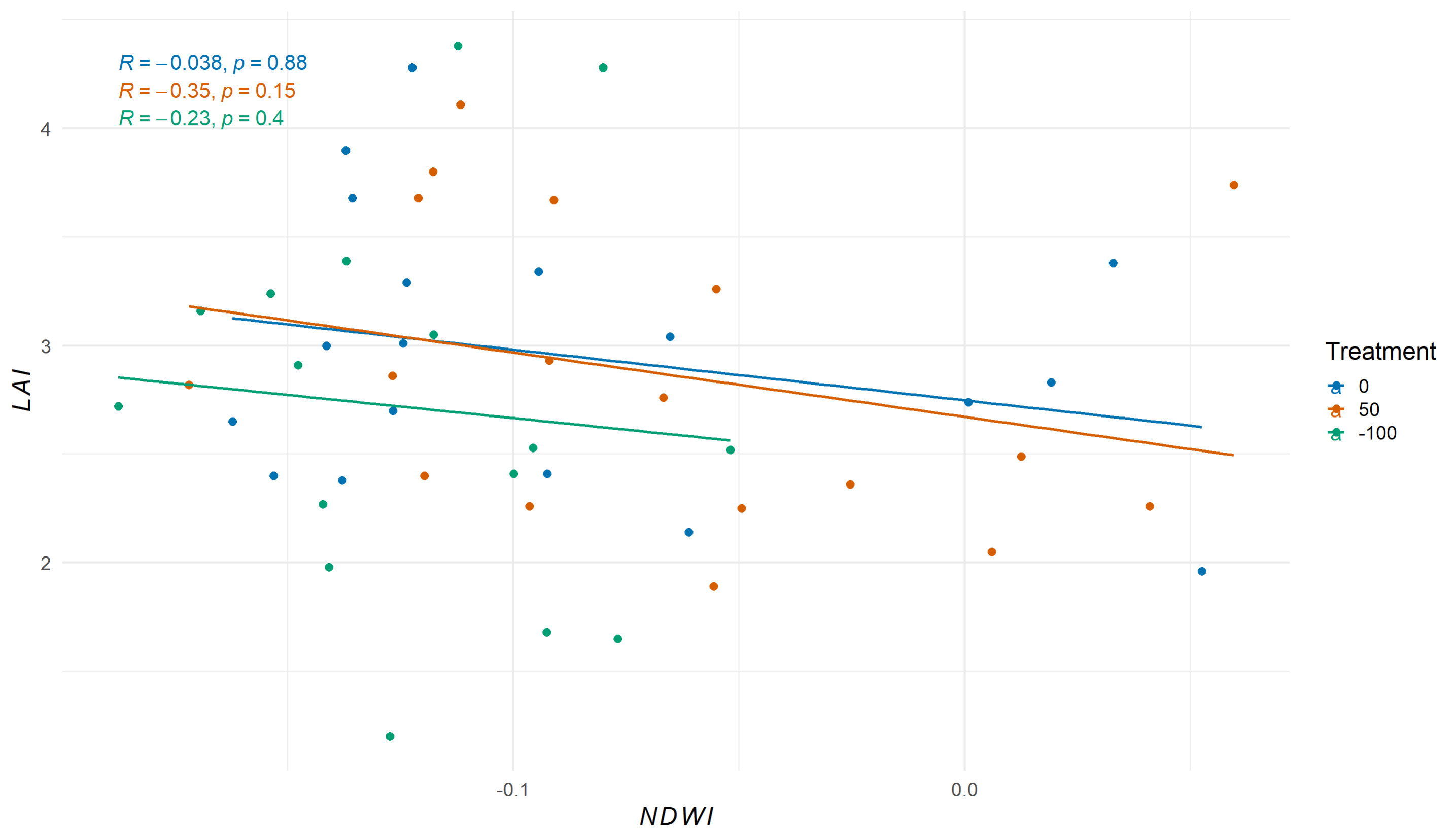
**

**Fig. S7.** A) Spearman correlations between LAI and CropScan NDVI interannually across all plots (N=21). B) Spearman correlations between LAI and NDVI interannually across precipitation gradient extremes (N=9). C) Spearman correlations between LAI and NDWI interannually across precipitation gradient extremes (N=9).

(A)


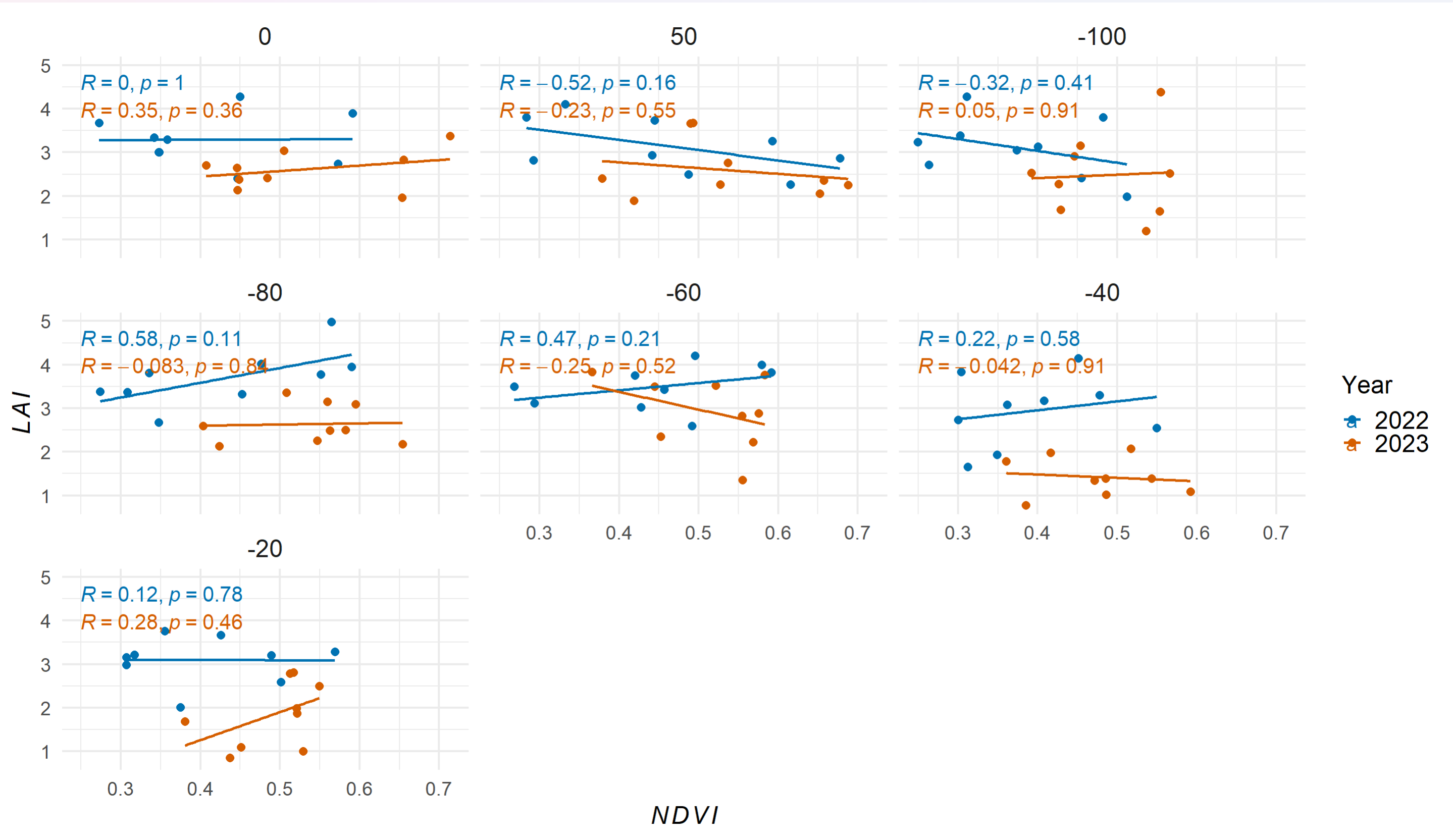


(B)

**
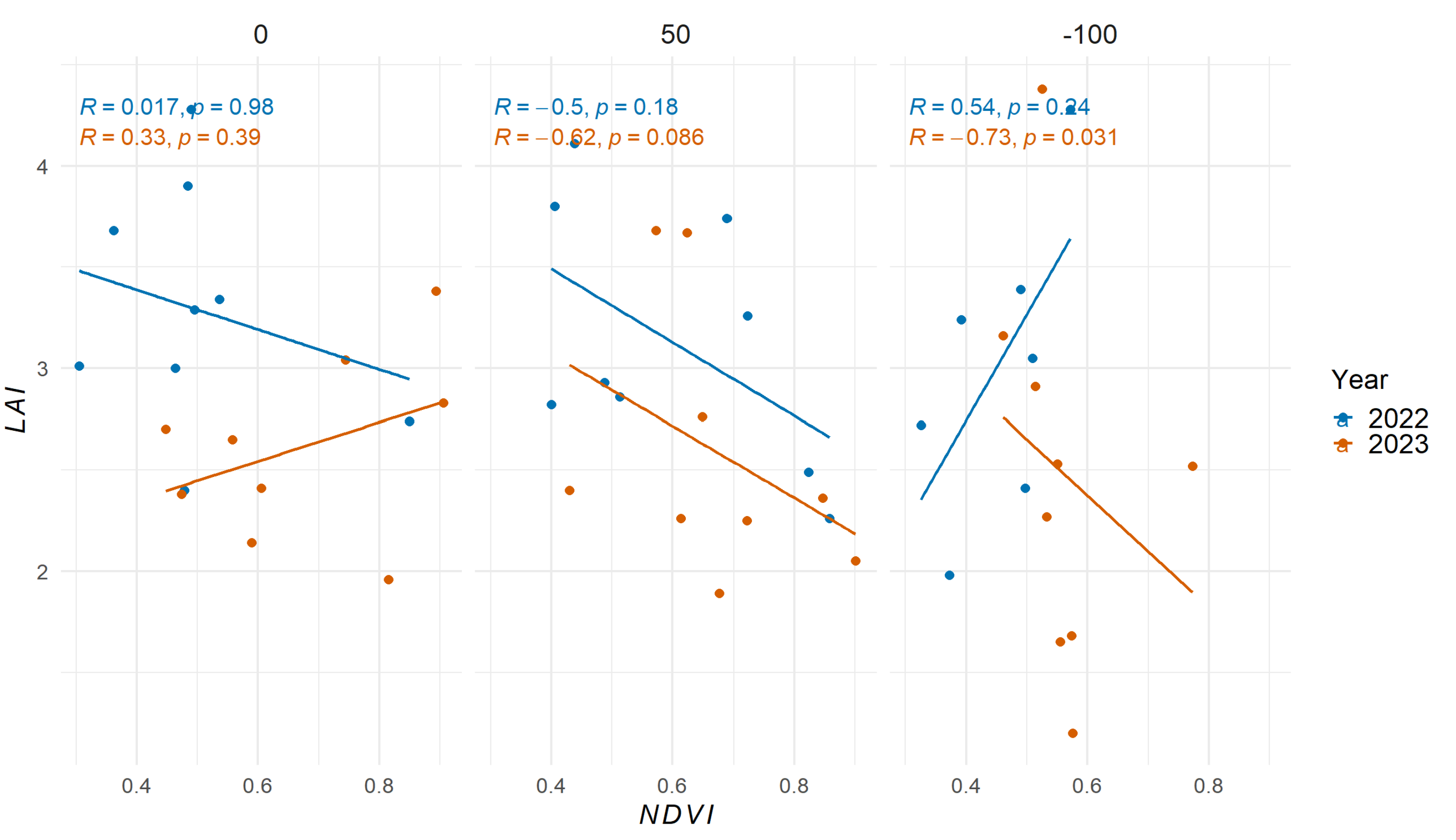
**

(C)


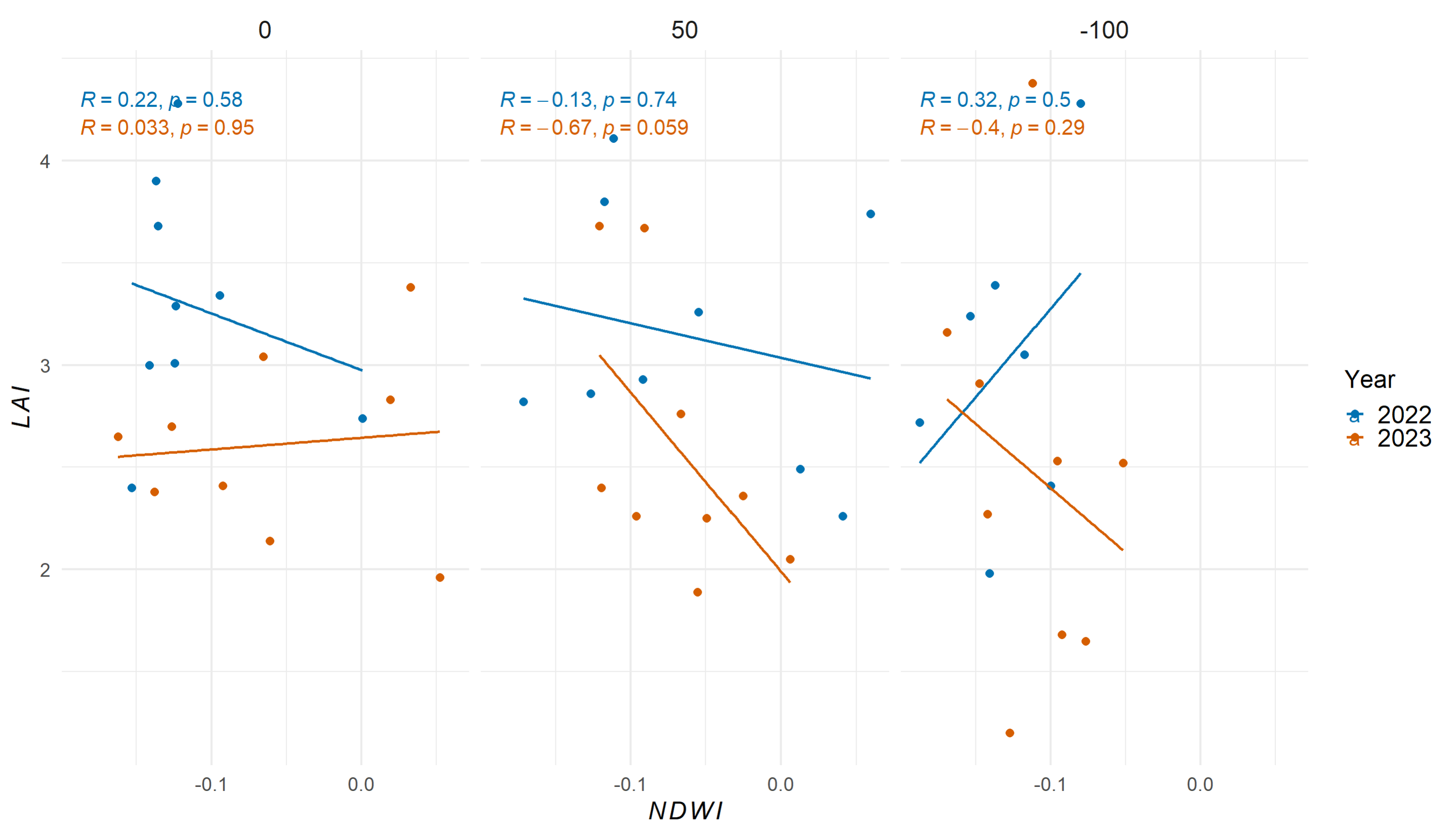


**Fig. S8.** A) Spearman correlations between LAI and CropScan NDVI showing seasonal variation across the precipitation gradient extremes (N=21). B) Spearman correlations between LAI and NDVI showing seasonal variation across the precipitation gradient extremes (N=9). C) Spearman correlations between LAI and NDWI showing seasonal variation across the precipitation gradient extremes (N=9).

(A)


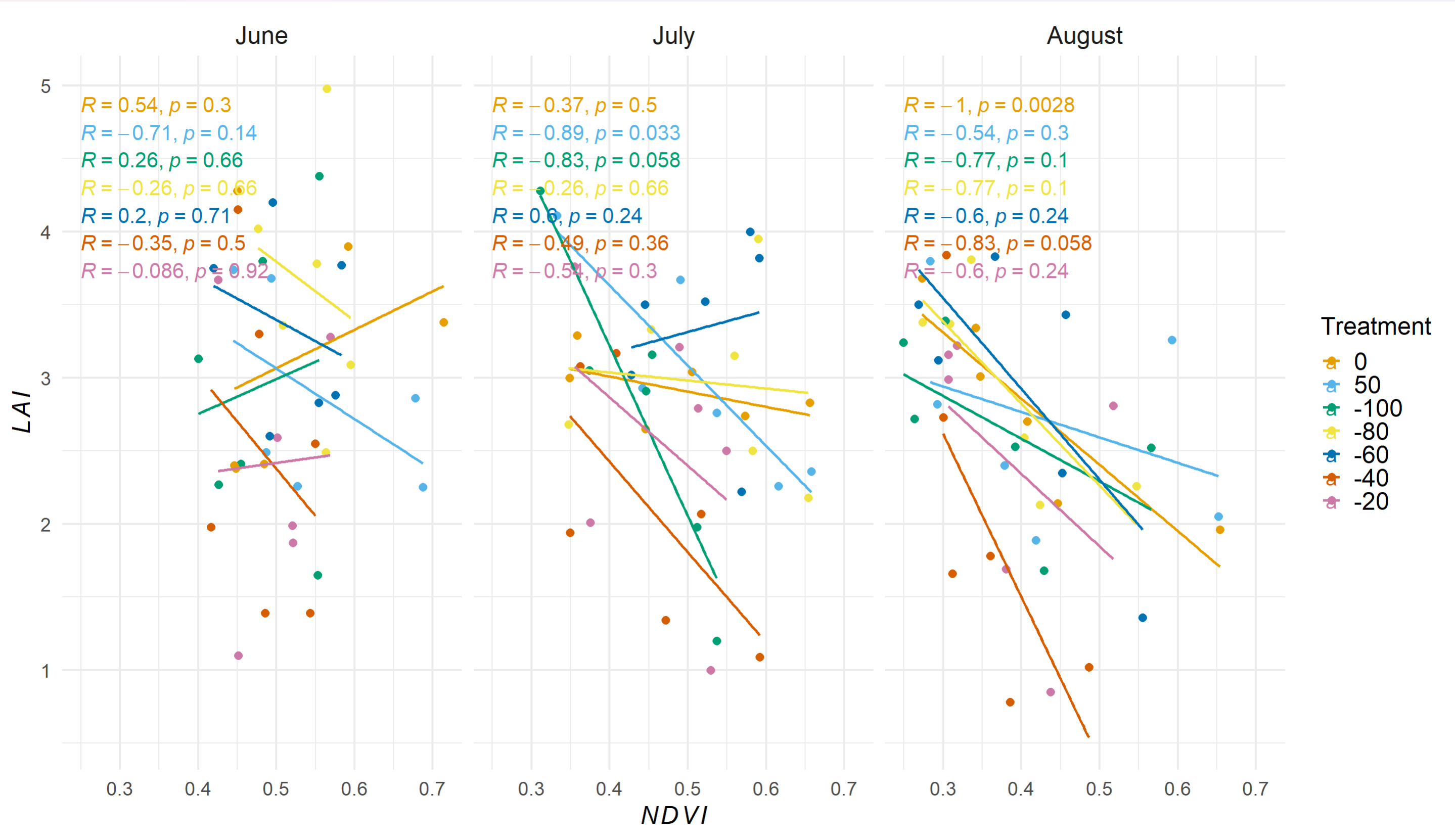


(B)


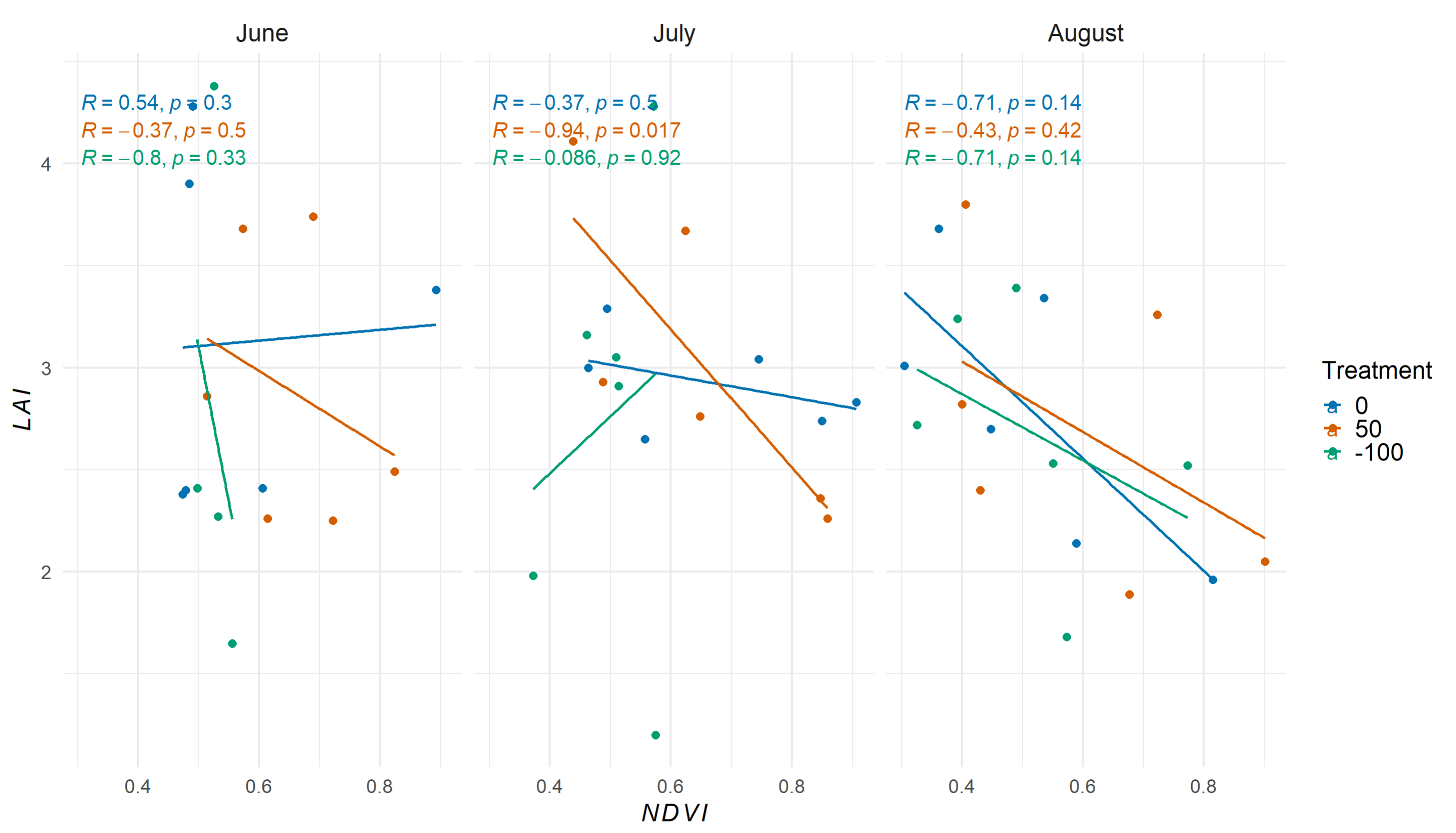


(C)

**
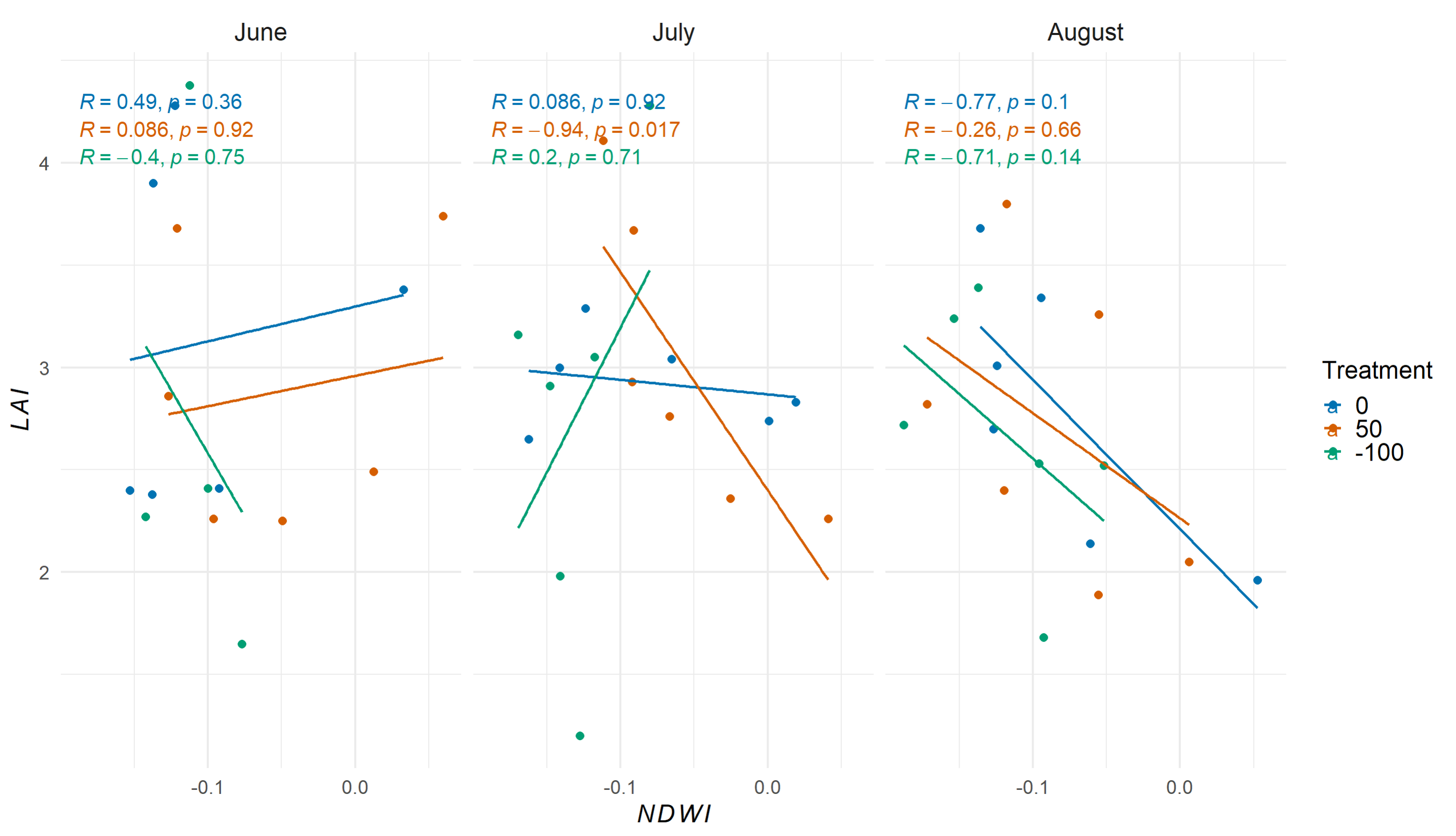
**
